# A comprehensive overview of omics-based approaches to enhance biotic and abiotic stress tolerance in sweet potato

**DOI:** 10.1093/hr/uhae014

**Published:** 2024-01-12

**Authors:** Sulaiman Ahmed, Muhammad Saad Shoaib Khan, Songlei Xue, Faisal Islam, Aziz Ul Ikram, Muhammad Abdullah, Shan Liu, Piengtawan Tappiban, Jian Chen

**Affiliations:** International Genome Center, Jiangsu University, Zhenjiang 212013, China; International Genome Center, Jiangsu University, Zhenjiang 212013, China; Jiangsu Coastal Area Institute of Agricultural Sciences, Yancheng 224000, China; International Genome Center, Jiangsu University, Zhenjiang 212013, China; International Genome Center, Jiangsu University, Zhenjiang 212013, China; Department of Plant Science, School of Agriculture and Biology, Shanghai Jiao Tong University, Minghang, 200240, Shanghai, China; International Genome Center, Jiangsu University, Zhenjiang 212013, China; Institute of Molecular Biosciences, Mahidol University, Nakhon Pathom, 73170, Thailand; International Genome Center, Jiangsu University, Zhenjiang 212013, China

## Abstract

Biotic and abiotic stresses negatively affect the yield and overall plant developmental process, thus causing substantial losses in global sweet potato production. To cope with stresses, sweet potato has evolved numerous strategies to tackle ever-changing surroundings and biological and environmental conditions. The invention of modern sequencing technology and the latest data processing and analysis instruments has paved the way to integrate biological information from different approaches and helps to understand plant system biology more precisely. The advancement in omics technologies has accumulated and provided a great source of information at all levels (genome, transcript, protein, and metabolite) under stressful conditions. These latest molecular tools facilitate us to understand better the plant’s responses to stress signaling and help to process/integrate the biological information encoded within the biological system of plants. This review briefly addresses utilizing the latest omics strategies for deciphering the adaptive mechanisms for sweet potatoes’ biotic and abiotic stress tolerance via functional genomics, transcriptomics, proteomics, and metabolomics. This information also provides a powerful reference to understand the complex, well-coordinated stress signaling genetic regulatory networks and better comprehend the plant phenotypic responses at the cellular/molecular level under various environmental stimuli, thus accelerating the design of stress-resilient sweet potato via the latest genetic engineering approaches.

## Introduction

Sweet potato (*Ipomoea batatas* [L.] Lam.) is the third most important storage root crop in the world after potato and cassava, with an annual production of approximately 113 million tons [1]. Sweet potatoes have become in widespread demand due to their high nutritive value, carbohydrate content, and health-promoting secondary metabolites [2]. Sweet potatoes possess a remarkable ability to grow in a wide range of environmental conditions [1] and have grown in many countries around the globe, such as tropical and sub-tropical countries in Asia, Africa, and Latin America [3]. Due to high adaptability to environments, sweet potatoes are primarily grown in arid, hilly regions with marginal soil conditions and exposed to various abiotic stresses. Recent climate change has also been a rising threat to global crop production; hence, the abiotic stresses to crop cultivation are becoming more severe and frequent, impairing plant growth development and reducing crop yield. Sweet potato production is also constrained by various biotic stresses, which cause typical sweet potato diseases and significantly threaten sweet potato quality. The co-evolution of sweet potatoes with different biotic diseases and their potato-microbe interaction study will be advantageous in improving the efficiency of breeding new biotic stress-resistant cultivars. The plants, being sessile, acclimatize to changes in environmental conditions and have developed various mechanisms to combat biotic and abiotic stresses. Thus, biotic and abiotic stress tolerance is necessary for sustainable sweet potato production.

Improving the crop traits is highly desired for developing superior varieties to deal with climate change and associated biotic and abiotic stress challenges. Biotic and abiotic stress tolerance mechanisms are complex, influenced by many external factors, and involve many cellular processes [4]. Climate change-driven global warming can trigger higher insect pest pressures and plant diseases, thus affecting sweet potato production sternly and influencing the storage root yield and quality [5, 6]. The sweet potato plant has evolved various anatomical, morphological, and metabolic adaptations under biotic/abiotic stresses [7, 8]. Stress signaling in sweet potatoes generally begins from energy sensing via many players, e.g., receptors, secondary messengers, transcriptional actors, regulatory enzymes, stress-responsive proteins, reactive oxygen species (ROS), and different organic–inorganic molecules involved in these complex processes [9]. Various transcription factors (TFs) play crucial roles in generating the earliest response under any stressful condition by acting as coordinators for signal transmission and controlling the regulation of stress-responsive genes. To date, the role of many TF families has been reported in regulating plant stress responses [10]. Understanding how sweet potato plants deal with different stresses helps to develop strategies for breeding high-productivity and high-quality sweet potatoes.

Crop breeding is a decision-making process where plant breeders select the individual plants harboring the best traits. Conventional breeding has been widely applied in sweet potato via hybridization and then subsequent rounds of selection, but these practices mostly remain very slow and inadequate to enhance the development of sweet potato varieties. The whole process takes multi-year testing at different locations to detect the genetic potential of candidate genotypes across a wide range of environmental conditions [11]. The development and application of molecular markers in sweet potato facilitated this process to some extent for the identification of desirable gentotypes [12]; however, improving the quantitative traits is quite challenging because they are controlled by numerous quantitative trait nucleotides. The traits controlling the genes for disease or stress tolerance are economically imperative in crop plants, therefore the extensive exploration of available germplasm resources and unraveling their genetic diversity remains vital for stress breeding programs. The advanced molecular breeding approaches provide unprecedented opportunities to accelerate the development of cultivars with desired traits and enhanced adaptation to mitigate the effects of climate change. With the advancement in genome sequencing and the latest genome editing technologies, the discovery and accumulation of valuable traits in a single genotype can now be done more efficiently. These innovative technologies provide a better understanding of genome structures and underlying trait architectures for precise crop improvement. The recent development in high-throughput next-generation sequencing technology during a couple of decades has led to the advent of ‘omics’ technologies: genomics, proteomics, transcriptomics, metabolomics, and phenomics. The integrated use of different omics approaches called multi-omics, such as pan-omics and trans-omics, also provides deep insights into data analysis, visualization, and interpretation to conclude the mechanism of any biological process, especially stress-related cellular mechanisms of plant responses and their relationships with the environmental clues to producing a characteristic phenotype. The efficient combination of different molecular breeding approaches, from markers to recent genomic and post-genomic era technologies, provide a basis to develop a successful breeding strategy that would be helpful to understand better the complex genetic control of biotic/abiotic related genes and their underlying plant resistance to obtain resistant crop varieties [13]. The integration of transcriptomic and metabolomic data was successfully applied in sweet potatoes to identify the candidate genes and corresponding metabolites [14]. The genome-wide analysis of expression QTLs has also been applied and reveal the regulatory architecture of gene expression profile in sweetpotato [15]. These approaches also facilitate the prompt identification of putative genes and their corresponding loci associated with stress resistance. Furthermore, omics technology also guides us in exploring stress-related pathways, which further helps plant functional and metabolic engineering. With the latest next-generation sequencing technologies, the extended information regarding the whole genome sequence and functional genomics resources facilitates stress breeding in tuber crops.

**Figure 1:**
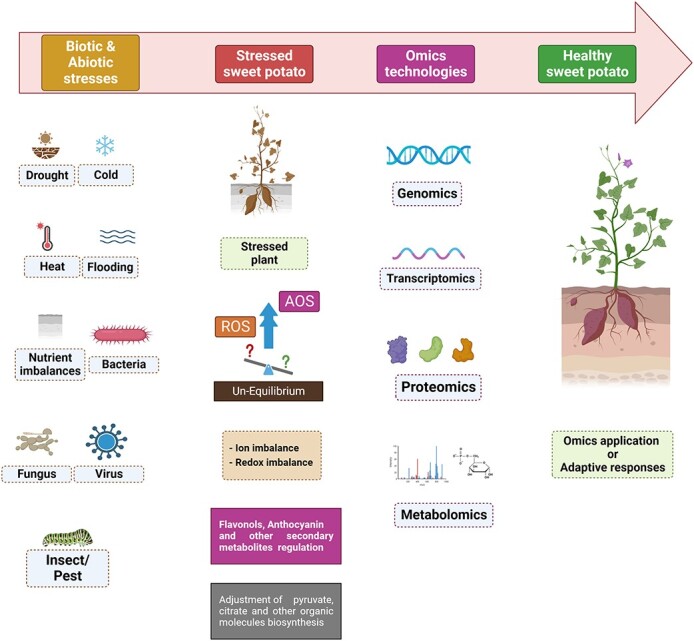
The use of the omics roadmap to develop stress-resilient sweet potato. Sweet potato usually grows on marginal lands, thus facing multiple biotic and abiotic stresses that cause considerable losses in sweet potato production worldwide. Recent genomics, transcriptomics, proteomics, and metabolomics approaches are used individually or in combination to understand the cellular mechanisms of sweet potato responses to multiple stresses, thus developing stress-tolerant sweet potato plants. AOS, alternative oxidase; ROS, reactive oxygen species.

In this review, we comprehensively describe the impact of major biotic and abiotic stresses on sweet potato, their adaptive mechanisms, and how the integration of different omics approaches such as functional genomic, transcriptomic, and proteomic processes enable the different researchers to combat the challenges of various diseases/pests as well as climate change posed by global warming. The updated consequences of major omics in sweet potatoes have been listed, and the impact/mechanism of the individual and multiple biotic and abiotic stresses have been drawn, which helps to explore the genetic regulatory networks for stress tolerance. It is important to identify the various component traits contributing to the stress tolerance mechanism, investigate the relative importance of these traits in various crops and production systems, and understand the genetic architecture of these component traits. The integrated use of various omics approaches in sweet potatoes helps design crops that perform better under environmental stresses and assist future sweet potato breeding for disease and environmental-stress-resilient cultivars (Fig. 1).

## Sweet potato whole genome sequencing and re-sequencing

High-throughput sequencing technology eases the way for whole-genome sequencing in different crops. An enormous amount of sequencing information is gathered as per the continuous reduction in sequencing cost per unit. However, there is limited information regarding whole-genome sequencing for sweet potatoes due to the highly heterozygous hexaploid genome of *I. batatas*, which further complicates genetic studies. Due to these limitations, sweet potatoes need further information and more sophisticated modern technologies that can process all the available information and draw a better perspective to explore the molecular mechanisms for biotic and abiotic tolerance in sweet potatoes. The first whole-genome *de novo* sequencing in diploid relatives of sweet potato (*Ipomoea trifida*) was reported in 2015 [16], facilitating the research in sweet potato studies as first reference genomes to better understand the stress resilance [17]. Furthermore, bacterial artificial chromosome library was constructed by using a stem nematode resistance line ‘Xu-781’, which provides a valuable source of polymorphic markers in the form of repetitive DNA sequences called simple sequence repeats (SSRs) for disease resistance and gives deep insights into the diploid closest wild relative of sweet potato genome (*I. trifida*) composition and their utilization in gene cloning and marker development [18]. Furthermore, the expression pattern of sporamin, which account for 80% of the total protein content in sweet potato, was also highlighted and its enzymatic activities were found to be related to plant defenses such as insects, plant diseases, and abiotic stresses (osmotic stress) [19]. The first chromosome-level references genome was constructed in wild relatives of sweet potato: *Ipomoea trifida* and *Ipomoea triloba* [20]. Yan *et al.* sequence the chloroplast genome in the sweet potato cultivar ‘Xushu18’, which facilitates and signifies the roles of chloroplast-related genes in response to future stress-responsive gene expression [21]. The whole-genome *de novo* assembly of carotenoid-rich (role as antioxidants during plant stresses) sweet potato cultivar was drawn, which provided helpful information regarding the complexity of chromosome sequence composition in a polyploidy genome [22]. The OutcrossSeq strategy was also applied in sweet potatoes to dissect loci for complex quantitative traits and identify several candidate genes for stress-related agronomic traits in sweet potato [23]. The re-sequencing of 314 sweet potato germplasm reveals several novel significant loci (Iba_chr02a, Iba_chr05a, Iba_chr06a, Iba_chr07a, Iba_chr10a) associated with stress-tolerance mechanisms related pathways such as carotenoid metabolism and anthocyanin metabolism [24]. The accessibility of this reference genome information permits its application in different polyploidy crops, and such technologies in sweet potatoes predicted significant progress and sped up the future precision breeding program for abiotic tolerance.

## Genome-wide survey of TF families and their response to various stresses in sweet potato

Different genome-wide surveys were conducted in sweet potatoes to identify TF families and their corresponding roles/expression under various stresses. Recently, a combined approach was adopted to study both biotic and abiotic stress responses in sweet potatoes and identified the role of *IbPIF3.1* TF under drought and *Fusarium* wilt stresses [25]. The comparative genome-wide study was performed between four *Ipomoea* species to evaluate valine glutamine motif-containing genes and found 40 differentially expressed genes (DEGs) referring to various abiotic stresses [26]. The other genome-wide studies in different *Ipomoea* species were conducted for important TF families such as NAC (*NAM*, *ATAF*, and *CUC*) TFs family [27], hydroxycinnamate-CoA quinate hydroxycinnamoyl transferase gene family [28], DNA-binding with one finger (Dof) TF family [29], myeloblastosis (MYB) gene family [30], phytochrome-interacting factors [25], domain of unknown function gene family [31], two-component system genes [32], xyloglucan endotransglucosylase/hydrolase gene family [33], expansin gene family [34], glycine-rich RNA-binding proteins [35], WRKY TFs family [36], zinc-finger domain-containing stress-associated proteins [37], plant-specific GRAS TFs [38], APETALA2/ethylene responsive factor (AP2/ERF) TFs family [38], jasmonate-ZIM (JAZ) TF family [39], and bZIP TF family [10]. These studies provide new insights for understanding the different TFs families-mediated stress responses and lays the foundation for future functional investigation of sweet potato TFs families.

**Figure 2:**
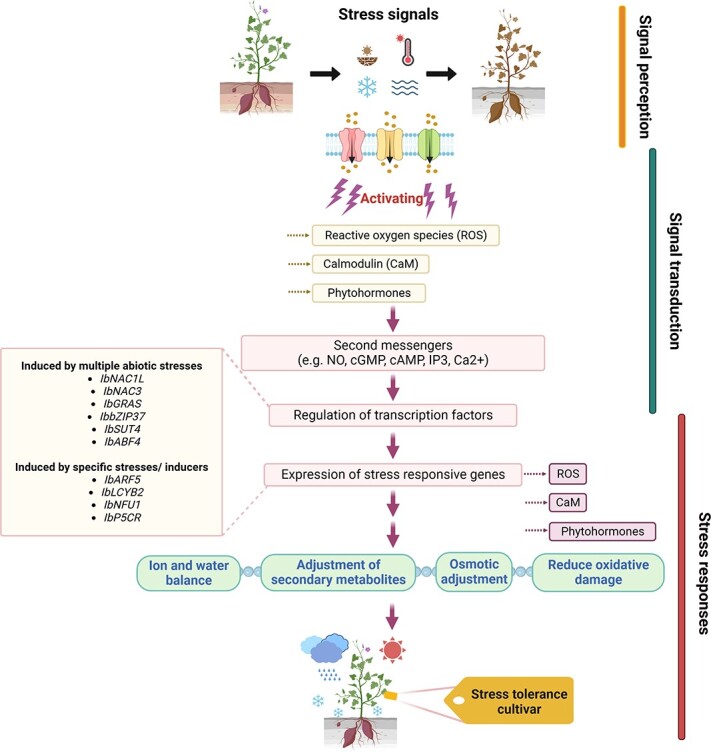
General overview of stress signaling pathway in sweet potato. Sweet potato biotic and abiotic stress signaling pathways are initiated by signal perception and lead toward stress responses. The early occurrence of stress sensing via receptors/sensors cascades activates the downstream stress-responsive genes via reactive oxygen species (ROS), Ca^2+^/calmodulin (CaM), and phytohormone signaling. Moreover, the signal transduction is facilitated by secondary messengers such as nitric oxide (NO), cyclic adenosine monophosphate (cAMP), cyclic guanosine monophosphate (cGMP), inositol triphosphate (IP3), and calcium ion (Ca^2+^). These signaling pathways induce the differential regulation of transcription factors (TFs) and other stress-responsive genes. Furthermore, regulating TFs/genes leads to adjusting sweet potato physiological, biochemical, and molecular responses, thus improving stress tolerance. The boxes on the left side indicate the essential genes/TFs that act and protect sweet potato plants under extreme environmental and biotic stresses.

**Figure 3:**
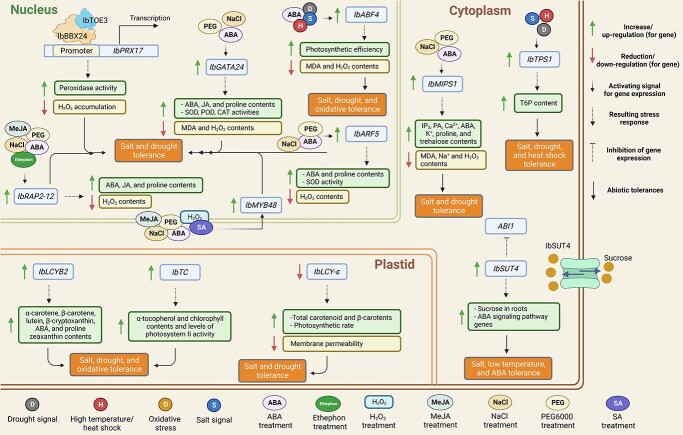
The molecular mechanism of sweet potato responses to multiple abiotic stresses. Sweet potatoes’ salt, drought, and oxidative stresses induce various transcription factors (TFs) in the nucleus. The B-box (BBX) TF IbBBX24 directly binds to the promoter of sweet potato peroxidases (*IbPRX17*) and stimulates the *IbPRX17* expression, leading to enhanced peroxidase activity along with the lower H_2_O_2_ accumulation [43]. A novel GAGA gene, *IbGATA24* (a kind of transcription factor), induces the expression by polyethylene (PEG) 6000, NaCl, and abscisic acid (ABA) treatments found in salt and drought tolerance [44]. Under salt and drought stresses, sweet potato with high *IbGATA24* expression shows increases in ABA, jasmonic acid (JA), and proline contents as well as high activities of superoxide dismutase (SOD), peroxidase (POD), and catalase (CAT) [44]. In addition, it is found that low contents of malondialdehyde (MDA) and H_2_O_2_ ultimately result in salt and drought stresses [44]. The sweet potato *IbABF4* encoding the ABA-responsive element binding factor is induced in its expression by ABA, drought, salt, and high-temperature signals leading to an increase in the photosynthetic efficiency and a decrease in the contents of MDA and H_2_O_2_ against salt, drought, and oxidative stresses [45]. Moreover, tolerance of sweet potato to salt and drought is involved in the up-regulation of *IbARF5*, *IbRAP2–12*, and *IbMYB48*. PEG6000, ABA, and NaCl trigger the up-regulation of *IbARF5* contributing to an increase in ABA and proline contents as well as the SOD activity along with the reduction of H_2_O_2_ [46]. Meanwhile, *IbRAP2–12* expression is induced by NaCl, PEG6000, ABA, and methyl jasmonate (MeJA), causing increases in ABA, JA, and proline contents with the reduction of H_2_O_2_ content [47]. The up-regulation of *IbMYB48* promotes the contents of ABA, JA, and proline as well as the SOD activity through the presence of PEG6000, NaCl, ABA, MeJA, SA, and H_2_O_2_ [48]. In plastid, *IbLCYB2* and *IbTC* play a significant role in salt, drought, and oxidative tolerance. The overexpression of lycopene β-cyclase gene *IbLCYB2* contributes to an increase in α-carotene, β-carotene, lutein, β-cryptoxanthin, ABA, and proline zeaxanthin contents [9], whereas the up-regulation of *IbTC* shows high accumulation of α-tocopherol and chlorophyll contents and high levels of photosystem II activity [49]. Additionally, the down-regulation of *IbLCY-ε* increases the contents of total carotenoid, β-carotene, and photosynthetic rate, thus reducing the membrane permeability, resulting in salt and drought tolerance [50]. In the cytoplasm, *Myo*-inositol-1-phosphate synthase gene (*IbMIPS1*) is induced by NaCl, PEG6000, and ABA and its overexpression significantly enhances salt and drought tolerance [64]. *IbMIPS1* up-regulation increases IP3, PA, Ca^2+,^ ABA, K^+^, proline, and trehalose contents whereas the reduction was observed in MDA, Na^+,^ and H_2_O_2_ contents [64]. Besides the up-regulation of trehalose-6-phosphate synthase (TPS), *IbTPS1* is induced by environmental stresses including, drought, salt, and heat shock, resulting in salt, drought, and heat shock tolerance with the increased trehalose-6-phosphate (TP6) content [77]. Moreover, a sucrose transporter plays a crucial role in plant growth and response to salt, low temperature, and exogenous ABA treatments. The overexpression of *IbSUT4* enhances sucrose accumulation in roots under various stress conditions as well as induces the expression of genes involved in ABA signaling pathways and inhibits the negative regulator of ABA signal transduction pathway gene expression, that is, *ABA insensitive 1* (*ABI1*) [63].

**Figure 4:**
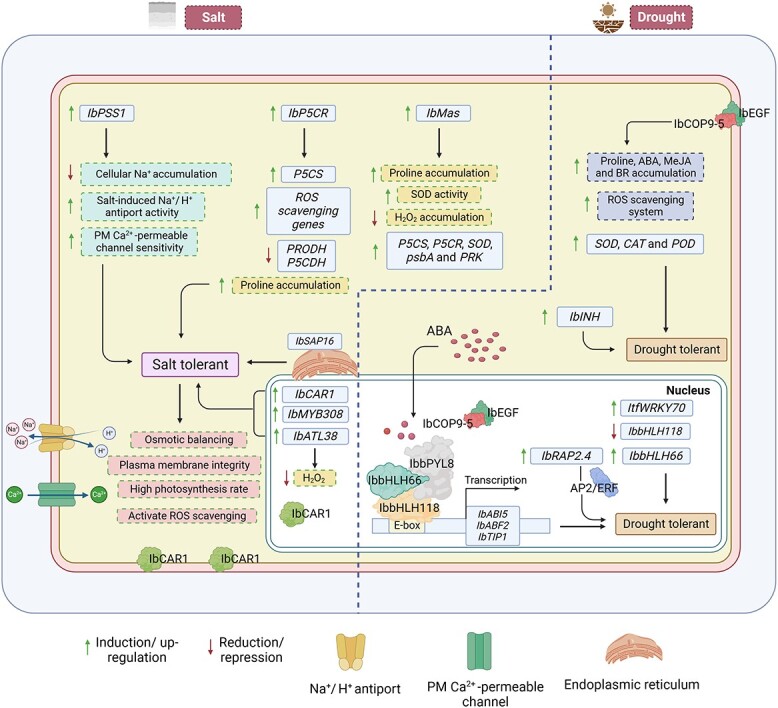
The molecular characterization of sweet potato responses under salt and drought stresses. Salinity is a genetically complex abiotic stress affected by several physiological and biochemical processes. Several physiological mechanisms that improve salt tolerance include ion exclusion from roots, ion compartmentalizing into vacuoles, regulation of ion transport from root to shoot, accumulation of organic compatible solutes in tissues, and increased tissue tolerance to toxic ions [70]. During salt stress, proline accumulation is common in plants. *Pyrroline-5-carboxylate reductase* (*P5CR*) is a critical enzyme in proline biosynthesis and is strongly induced by salt stress [66]. The overexpression (OE) of *IbP5CR* stimulates the expression of ROS scavenging genes, whereas it down-regulates the *proline dehydrogenase* (*PRODH*) and *P5C dehydrogenase* (*P5CDH*) genes and enhances proline accumulation [66]. The OE of sweet potato *Maspardin* gene (*IbMas*) contributes to an increase in both proline accumulation and superoxide dismutase (SOD) activity and decreases H_2_O_2_ accumulation. Moreover, *IbMas* expression also causes the up-regulation of *P5CS*, *P5CR*, *SOD*, *PsbA* (D1 protein of PSII), and *Phosphoribulokinase* (*PRK*) genes [67]. Sweet potato phosphatidylserine synthase-1 (*IbPSS1*) encodes the enzyme involved in phosphatidylserine (PS) synthesis [68]. Under salt stress, an apoplastic H_2_O_2_ burst is rapidly triggered [71]. The OE of *IbPSS1* causes a reduction in cellular Na^+^ accumulation, but also elevates salt-induced Na^+^/H^+^ antiport activity and plasma membrane (PM) Ca^2+^ permeable channel sensitivity to NaCl and H_2_O_2_ [68]. The sweet potato stress-associated protein (SAP) gene *IbSAP16* is present in the endoplasmic reticulum and responds under salinity stress [37]. Sweet potato C2-Domain Abscisic Acid-Related Gene *IbCAR1* [72], RING-H2 type E3 ubiquitin ligase gene *IbATL38* [73], and APETALA2/ethylene responsive factor (AP2/ERF) transcription factor (TF) *IbRAP2.4* [74] and Myeloblastosis (MYB) TF *IbMYB308* [69] play significant roles in salinity stress. The two bHLH proteins have antagonistic roles in ABA-mediated drought responses. IbbHLH118 directly binds to the promoter of *IbABF2* (*ABA-responsive element binding factor 2*), *IbABI5* (*ABA-insensitive 5*), and *IbTIP1* (*Tonoplast intrinsic protein 1*) at the E-box region to suppress their transcription. ABA accumulates during drought stress, promoting the IbPYL8–IbbHLH66–IbbHLH118 complex formation, repressing the ABA-responsive genes, and improving drought tolerance [62]. An open reading frame (sORF) gene in sweet potato [75], *IbEGF*, and sweet potato phytochrome-interacting factors (*IbPIF3.3*) also play significant roles in drought tolerance [25, 75]. *IbEGF* protein is present in the nucleus and cell membrane and it interacts with the *IbCOP9-5α* and enhances drought tolerance by increasing the accumulation of proline, ABA, methyl-jasmonate (MeJA), brassinosteroid (BR) and activating ROS scavenging system as well as up-regulating genes involved in SOD, catalase (CAT), and peroxidase (POD) [75].

## The functional genomics studies for resilience against abiotic and biotic stresses in sweet potato

Functional omics data from multiple platforms are useful for positional cloning. The stress signaling in sweet potatoes generally evolves by signal perception, which then leads toward the stress responses. Many players from receptors, regulatory enzymes, and different organic–inorganic molecules are involved in this complex process. Signal extension and transduction by secondary messengers cause differential regulation of TFs and stress-responsive genes (Fig. 2). The plant functional omics data are helpful for positional cloning and utilized to unravel the various stress-related molecular mechanisms, thereby contributing to the development of functional genomics for stress tolerance in tuber crops [40].

### Role of TF families and other resistance genes incorporating stress resistance

The advancement in functional genomics has accelerated the studies to dissect the complex role of TFs in biotic and abiotic stress tolerance mechanisms and led to the identification of novel candidate genes/alleles. Different plant-specific TF families such as NAC, MYC/MYB, c-repeat binding factor (CBF)/dehydration response element binding (DREB), ABA-responsive elements (ABRE), and AP2/ERF regulate multiple abiotic stress-related gene expression. NAC family TFs function in different regulatory networks in response to numerous abiotic stresses. Recently, *IbNAC3* TF was found as a crucial component for incorporating combined stress tolerance via integrating different regulatory events as well as ubiquitin pathways [41]. A dynamic network biomarker analysis was performed on sweet potato and identified *IbNAC083* as a core regulator and its gene ontology (GO) enrichment revealed most of the stress and hormonal-responsive genes [42]. In another study, sweet potato *NAC006* and *NAC143* had prominent transactivation activities and were strongly induced by multiple abiotic stress (Fig. 3) [27]. The gene expression analysis of 12 novel *NAC* genes (designated as *IbNAC1L* and *IbNAC3* through *IbNAC13*) were significantly induced by implying multiple abiotic stresses as well as when treated with various hormones, depicting distinct roles of *IbNAC* genes in sweet potato stress tolerance [51]. The *IbNAC1*-overexpressing (OE) sweet potato plants increased the sporamin expression and elevated resistance against herbivores via the jasmonic acid (JA)-mediated pathway [52]. Furthermore, nucleotide-binding sites and leucine-rich repeat domains containing genes are plants’ predominant type of resistance genes. Four NBS encoding genes’ (*IbNBS258*, *IbNBS88*, *IbNBS10*, and *IbNBS20*) expression were significantly induced in response to stem nematode infection in sweet potato [53].

The role of a basic helix–loop–helix (bHLH) TF *IbbHLH33* was reported as a positive regulator in cold tolerance and fine-tune signaling pathways for chilling stress [54]. In another study, Zhang *et al.* [55] reported the role of 72 putative sweet potato *IbGRAS* genes in responses to multiple abiotic stresses (salt, drought, heat, and cold). Three sweet potato expansin (*IbEXP1*, *IbEXP2*, and *IbEXPL1*) activities were found significant under chilling stress, and their transcriptional regulation provides a way forward to enhance the chilling tolerance in tropical crops [56]. Moreover, sweet potato zinc finger protein gene-1 (*IbZFP1*) develops drought and salt tolerance in transgenic *Arabidopsis* plants via modulating abscisic acid (ABA) signaling, ROS scavenging, proline biosynthesis, and stress-responsive genes [57]. The functional study of a PIFs TFs *IbPIF3.1* revealed that *IbPIF3.1* enhances drought and *Fusarium* wilt tolerance in transgenic tobacco plants and is significantly induced by salt, H_2_O_2_, cold, and heat stresses [25].

### Role of sweet potato secondary metabolic pathways to mitigate stress responses

The role of plant secondary metabolites is well known to cope with adverse environmental conditions. The genes involved in the carotenoid biosynthesis pathway were induced under abiotic stress responses. A recent report suggested the role of *IbNAC29* in carotenoid accumulation via inducing the expression of carotenoid biosynthesis gene *IbPSY* and directly binds to sweet potato STAY-GREEN-1 (*IbSGR1*) and inhibits the promoter activity of *IbSGR1* [58]. Under salt stress, *IbOr-Ins* OE plants showed increased carotenoid contents, which is closely associated with higher DPPH radical-scavenging activity [59]. In another report, *IbARF5* transgenic *Arabidopsis* plants showed increased carotenoid contents and enhanced tolerance to salt and drought stresses [46]. The overexpression of sweet potato *lycopene beta-cyclase* (*IbLCYB2*) gene enhances the drought, salt, and oxidative stress tolerance via increasing the contents of α-carotene, β-carotene, β-cryptoxanthin, lutein, and zeaxanthin [9]. Moreover, down-regulation of *CHY-β* and *LCY-ε* genes showed tolerance to salt stress compared to wild-type control cells and calli of sweet potato [60]. Anthocyanin’s natural pigments belong to the main subgroup of flavonoids, which not only gives plants distinguishable colors but also provides them with resistance against biotic and abiotic stresses. The transcriptomics data screening from rich anthocyanin cultivars identified a R2R3-MYB gene *IbMYB48*, for which the expression is induced by various stresses, and enhances the drought and salt tolerance in sweet potatoes [48]. In another study, the down-regulation of sweet potato dihydroflavonal-4-reductase gene *IbDFR* increases susceptibility to abiotic stress while decreasing the anthocyanin accumulation [61].

### The hormonal interplay to incorporate stress resilience in sweet potato

ABA is widely known for its pivotal roles in responding to various abiotic stresses and regulating plant adapting metabolism and gene expression profiles to cope with stress responses. The antagonistic roles of two *bHLH* were established under ABA-mediated drought responses; that is, OE of *IbbHLH118* enhances drought susceptibility, whereas the OE of *IbbHLH66* increases drought tolerance [62]. In another study, sucrose transporter gene *IbSUT4* behaves through the ABF-dependent ABA signaling pathway and plays critical roles under abiotic stresses [63]. ABA-induced expression of *IbbZIP37* and *IbMIPS1* under various environmental stresses thus enhances the salt and drought tolerance under field conditions [64, 65]. The OE of different sweet potato genes, i.e., *IbSPCP2*, *IbNFU1*, *IbP5CR*, *IbMas, IbPSS1,* and *IbMYB308*, significantly enhances salt and drought tolerance in transgenic plants by protecting membrane integrity, regulating osmotic balance, photosynthesis, maintaining Na^+^ homeostasis, Na^+^ exclusion in the root, and activating the ROS scavenging system [66–69] (Fig. 4). The JAZ domain is a core JA-signaling module that regulates the expression of JA-responsive genes. The OE of the *IbBBX24* gene increases the resistance against *Fusarium* wilt disease via modulating the JA biosynthesis pathway via directly binding to the promoter region of JA signaling repressor *IbJAZ10* and activating its transcription. These findings suggested that JA responses play a crucial role in regulating *Fusarium* wilt resistance in sweet potato [76] (Table 1).

**Table 1:** A detailed overview of abiotic and biotic stress studies in sweet potato.

**Host cultivar**	**Resistance gene/ candidate gene/ target gene**	**Abiotic/biotic stresses**	**Defense mechanism/ catabolism/ signaling/ significant compounds involving stress tolerance**	**Ref.**
Xushu 18	Trehalose-6-phosphate synthase 1 (*IbTPS1*)	Salt, drought, and heat shock (47°C)	The increase in T6P content acting as a secondary messenger for inducing the expression of genes related to signal transduction in response to abiotic stresses	[77]
HVB-3; Shangshu 19	Lycopene β-cyclase (*IbLCYB2*)	Salt, drought, and oxidative stresses	The upregulated genes involved in carotenoid and ABA biosynthesis pathways	[9]
Xushu 29	Tocopherol cyclase (*IbTC*)	Salt, drought, and oxidative stresses	The increases in photosynthesis II activity, chlorophyll, and α-tocopherol content contents	[49]
Yulmi	Lycopene ε-cyclase (*IbLCY- ε*)	Salt and drought	The increases in carotenoid and ABA accumulation, β-carotene, and ROS	[50]
ND98 (salt-tolerant); Lizixiang (salt-sensitive)	The B-box (*IbBBX24*) and peroxidase (*IbPRX17*)	Salt and drought	The higher peroxidase activity along with lower H_2_O_2_ accumulation	[43]
Xushu55-2	*IbGATA24*	Salt and drought	Genes involved in ABA and JA signaling pathways and ROS scavenging were upregulated IbGATA24 binding with IbCOP9-5a protein through the activation of ABA, and ROS accumulation enhanced salt and drought stress resistance	[44]
Yulmi; Sinhwangmi; Sinzami	Orange protein of sweet potato (*IbOr*) and *carotenoid cleavage dioxygenase 4* (*IbCCD4*)	High temperature (47°C) and drought	The increases in carotenoid contents	[78]
Sinzami; Yulmi;Sinhwangmi	*IbMPK3/6*	*Pseudomonas syringae* pv. *tabaci* and cold temperature	*IbMPK3/6* phosphorylation Upregulation of PR gene expression Upregulation of *IbMPK3/6* against cold stress	[79]
Nongda 603; Lizixiang	*IbMIPS1*	Salt and drought tolerance, and stem nematodes	Upregulation of genes involved in inositol biosynthesis, PI and ABA signaling pathways, stress responses, photosynthesis, and ROS scavenging system The increases in the contents of inositol. IP_3_, PA, Ca^2+^, ABA, callose, and lignin	[40, 64]
HVB-3	*IbARF5*	Salt and drought	The increases in the contents of carotenoids, ABA, proline, and SOD activity The reduction of H_2_O_2_ content The upregulation of genes related to carotenoid and ABA biosynthesis and abiotic stress response	[46]
ND98	*IbRAP2–12*	Salt and drought	The increases in the contents of ABA, JA, and proline with the reduction of H_2_O_2_ content The upregulation of genes involved in ABA and JA signalling, proline biosynthesis and ROS scavenging process	[47]
Xushu 55–2	*IbWRKY2*	Salt and drought	The increases in the contents of ABA and proline with the reduction of MDA and H_2_O_2_ contents The upregulation of genes involved in ABA signalling, proline biosynthesis, and ROS scavenging process	[80]
Xushu 18	*IbABF4*	Salt, drought, and oxidative stress	The increases in ABA sensitivity and photosynthetic efficiency with the reduction of MDA and H_2_O_2_ contents	[65]
Taizhong 6	*IbSUT4*	Salt, low temperature, and exogenous ABA	The reduction of MDA content A higher accumulation of sucrose content in roots and the lower sucrose content in leaves. The changes of sucrose distribution between source and sink tissues were responsible for salt, low temperature, ABA treatment	[63]
Jingshu 6 and JS6-5	*IbMYB48*	Salt and drought	The increases in ABA, JA, and proline contents with the enhance of SOD activity The upregulation of genes involved in ABA and JA biosynthesis, and ROS scavenging system	[48]
Xushu 18	Myo-inositol-1-phosphate synthase (*IbMIPS1*)	Salt stress	Salt stress-responsive genes including MIPP, P5CS, P5CR, PRK, and SOD were upregulated The contents of inositol, proline, SOD, and photosynthesis activities were increased	[81]
Whitestar	Peroxidase (*swpa4*)	Oxidative stress and high salinity	Peroxidase (swpa4) played a role in the regulation of peroxidase metabolism against stress responses Interplay among diverse peroxidases regulated stress responses	[82]
Yeonjami (YJM, tolerant); Jeonmi (JM, highly sensitive)	Ethylene response factor VII (*ERFVII*)	Flooding	Genes involved in ET, ROS, NO biosynthesis were upregulated *ERFVII*, a gene related to low oxygen signaling, was upregulated	[5]
Lushu 3	Trehalose-6-phosphate synthase (*IbTPS*)	Salt tolerance	High accumulation of trehalose, and proline contents	[83]
Xushu 29	Invertase inhibitor (*IbINH*)	Drought	The increases in sucrose content and upregulation of ABA biosynthesis genes were noticed during water deficit	[84]
Xushu55-2; Lizixiang	*IbEGF*	Drought	High accumulation of ABA, MeJA, BR, and proline contents and upregulation of genes encoding SOD, CAT, and POD were reported under stress conditions IbEGF-IbCOP9-5α interaction increased drought tolerance through the regulation of phytohormone signalling pathways	[75]
Xushu 29	Basic/helix-loop-helix (*IbbHLH79*, an *ICE1*-like gene)	Cold tolerance	Induction of an active C-repeat binding factor (CBF) pathway mediated a broad range of signals in sweet potato	[85]
Xushu 29	Lignin-forming peroxidase (*IbLfp*)	Low-temperature storage ability	The increases in POD activity and lignin accumulation Reduction of MDA and H_2_O_2_ contents	[86]
Xushu 29	*IbFAD8*	Low-temperature storage ability	The increases in ALA content, membrane fluidity and POD activity	[87]
Xushu 29	Orange gene (*IbOr-R96H*)	Heat tolerance (47°C)	The increases in the contents of carotenoid, Β-carotene, and ABA as well as the DPPH radical scavenging activity	[88]
G87 × N73 (F1)	*SPWR1* and *SPWR2*	Sweet potato weevil	Increase in quinate derivative metabolites	[89]
Guangshu No. 87	*IbPAL, IbC4H, and IbHQT*	Sweet potato weevil	The increases in JA, SA, and ABA The levels of *IbPAL*, *IbC4H*, and *IbHQT* expression were up-regulated	[90]
Kyushu No. 166; Tamayutaka	The terpenoid-related genes (*itf09g05600.t1*, *itf09g05580.t1*, and *itf12g13950.t1*)	Sweet potato weevil	The suppression of pupation during larval development An increase in the expression of terprnoid-related genes	[91]
Ayamurasaki	*Cry1Aa*	*Spodoptera litura*	*Cry1Aa* toxin expressing in BT transgenic sweet potato lines could prevent *S. litura* infestation *Cry1Aa* toxin severely damages the columnar cells of midgut of *S. litura*	[92]
Xushu29	*IbSPF1*	*P. syringae* pv. *tabaci*	*IbSPF1* interacted with *IbMPK3/6* and was phosphorylated at Ser75 and Ser110 by *IbMPK3/6* The increase in the affinity of IbSPF1 for W-box element	[93]
ND98; Lizixiang	*IbSWEET10*	*Fusarium oxysporum*	Sugar contents decreased in leaves and the remaining compact cells in the pith were observed	[94]
ND98; Lizixiang	*IbBBX24*	*F. oxysporum*	High accumulation of JA. JA biosynthesis and signaling played a significant role in *Fusarium wilt* resistance in sweetpotato	[76]
Tainong 57	*IbNAC1*	Wounding	A forming protein complex of IbbHLH3-IbbHLH3 binding the G-box motif to activate *IbNAC1* expression	[52]
Kokei No.14; Tamayutaka	α-hordothionin (αHT) from barley endosperm	*Ceratocystis fimbriata* (Black rot)	Plant thionin peptide had an anti-fungal activity against *C. fimbriata* Thionin inhibits the fungal growth at the inoculated site through the action at their membranes	[95]
Chikei 682–11	Coat protein (CP) gene of SPFMV	*Sweet potato feathery mottle virus* (SPFMV)	CP-mediated resistance	[96]
Blesbok	CP genes of SPFMV, *Sweet potato chlorotic stunt virus* (SPCSV), *Sweet potato virus G* (SPVG), and *Sweet potato mild mottle virus* (SPMMV)	Multiple viruses (SPFMV, SPCSV, SPVG, and SPMMV)	CP-mediated resistance manifested the delayed symptoms of chlorosis and mottling of lower leaves	[97]
15 sweet potato diallel progenies (1352 genotypes)	*spcsv1* and *spfmv1*	SPCSV and SPFMV	The recessive genes, *spcsv1* and *spfmv1* associated with the resistance to SPCSV and SPFMV, respectively	[98]
Huachano	An intron-spliced hairpin contruct targeting the RNA-dependent RNA polymerase (*RdRp*) encoding sequence of SPCSV	SPCSV	RNA silencing-mediated resistance	[99]
HZHK2	*chit42* from *Trichoderma harzianum*	*Sclerotinia sclerotiorum* (white rot)	The increase in the endochitinase activity caused a reduction of *S. sclerotiorum* virulence	[100]
Xushu 29	SPCSV-RNase3	Sweet potato virus disease (SPVD)	CRISPR-Cas13 system An *RNase3*-targeted RfxCas13d system improved the SPVD resistance	[101]

ABA, abscisic acid; ALA, alpha-linoleic acid; ARF, auxin response factor; BR, brassinosteroid; BT, *Bacillus thuringiensis*; CAT, catalase; C4H, cinnamate 4-hydroxylase; CRISPR-Cas, clustered regularly interspaced short palindromic repeat CRISPR-associated protein; DPPH, 2,2-diphenyl-1-picrylhydrazyl; ET, ethylene; GB, glycine betaine; H_2_O_2_, hydrogen peroxide; HQT, hydroxycinnamoyl-CoA quinate hydroxycinnamoyl transferase; inositol-1,4,5-triphosphate, IP3; JA, jasmonic acid; MDA, malondialdehyde; MeJA, methyl-jasmonate; NO, nitric oxide; PEG, polyethylene glycol; PI, phosphatidylinositol; POD, peroxidas; PR, pathogenesis-related; ROS, reactive oxygen species; SA, salicylic acid; SOD, superoxide dismutase.

### The activation of various stress-responsive signaling pathways/cascades during multiple stresses

**Figure 5:**
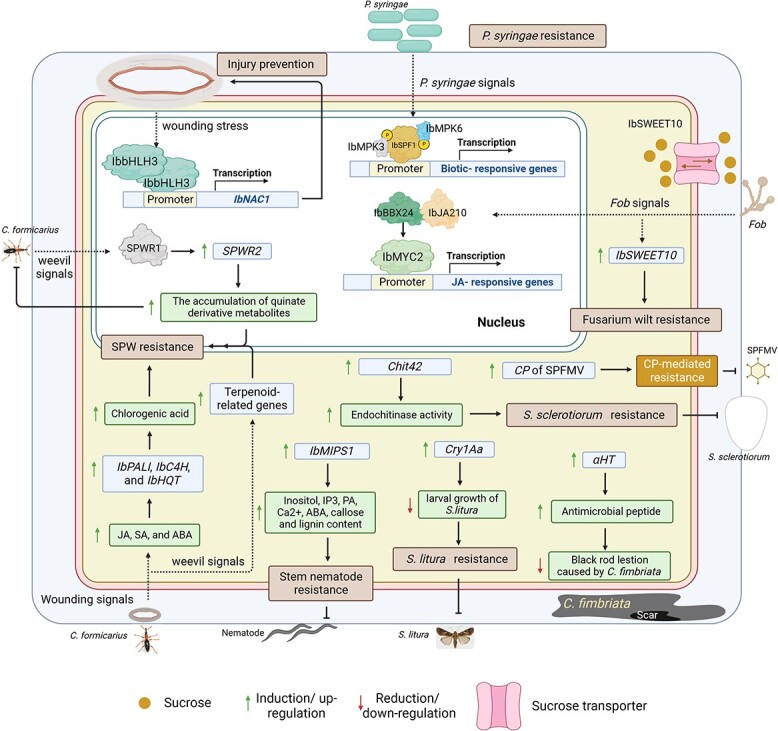
The cellular responses under biotic stress to incorporate tolerance mechanism in sweet potato. The fusarium wilt in sweet potatoes is caused by *Fusarium oxysporum* f. sp *batatas* (*Fob*). *Fob* infection induced the expression of a sucrose transporter gene *IbSWEET10*, which confers resistance to *Fob* infection [94]. Moreover, the IbBBX24 also plays a significant role in fusarium wilt resistance caused by *Fob* in sweet potato by competition with IbMYC2 to interact with *IbJAZ10* and enhance the function of IbMYC2 to activate jasmonic acid (JA) signaling, leading to *Fob* resistance [76]. Sweet potato SP8-binding factor (*IbSPF1*) acts as a transcriptional regulator of biotic stress signaling in sweet potatoes. IbSPF1 is phosphorylated by IbMPK3 and IbMPK6 at Ser75 and Ser110, respectively, which increases the binding affinity with the w-box element in target gene promoters, resulting in tolerance to *Pseudomonas syringae* [93]. The overexpression of *IbMIPS1* enhances the expression of genes involved in inositol biosynthesis, phosphatidylinositol (PI) as well as increases the content of inositol, inositol-1,4,5-trisphosphate (IP_3_), phosphatidic acid (PA), Ca^2+^, abscisic acid (ABA), callose, and lignin to protect stem nematode infection [64]. *IbNAC1* plays an important role in the defense responses against herbivores in sweet potatoes through the binding of the IbbHLH3-IbbHLH3 protein complex at G-box motif to activate *IbNAC1* expression [52]. In addition, *α-hordothionin* (*αHT*) encoding the antimicrobial peptide induces the expression in the fusion forms of *E12Ω:αHT* and *β-Amy:αHT* on sweet potato [95]. Storage roots of sweet potato harboring the *αHT* show the reduction of black rod lesion caused by *Ceratocystis fimbriata* [95]. Sweet potato weevils (SPWs) or *Cylas formicarius* are among the most significant pests, which cause substantial losses in sweet potato yield [89]. Wounding from SPWs activates the increases in phytohormones, jasmonic acid (JA), salicylic acid (SA), and abscisic acid (ABA) and subsequently induces the expression of genes involved in chlorogenic acid (*IbPAL*, *IbC4H*, and *IbHQT*) to enhance the accumulation of chlorogenic acid, resulting in resistance against SPWs [90]. A recent study reveals the SPW resistance through the functions of both *SPWR1* and *SPWR2* by activating the quinate biosynthesis [89]. Moreover, transcriptomics of SPW-resistant sweet potato, Kyushu No. 166 (K166), exhibits that terpenoid-related genes play an important role in SPW resistance [91]. *Spodoptera litura* is one of the crucial insect pests of sweet potato [92]. Feeding sweet potato leaves with overexpressing *Cry1Aa* on *S. litura* caused the reduction of larval growth by destroying its midgut, indicating that overexpressing *Cry1Aa* sweet potato can prevent *S. litura* infestation [92]. In addition, coat protein-mediated resistance to sweet potato feathery mottle virus (SPFMV) is reported by inducing the expression of the coat protein (*CP*) gene of SPFMV in sweet potato var. Chikei 682–11 [96]. The chitinase gene, *chit42*, from *Trichoderma harzianum* is introduced into sweet potato and promotes the activity of endochitinase, resulting in resistance to white rod disease caused by *Sclerotinia sclerotiorum* [100].

The stress-responsive signaling pathways/cascades significantly mitigate the detrimental factors posed by various stresses in plants. During multiple abiotic stresses, various signaling cascades in sweet potato activate and induce different TFs via signal transduction pathways and initiate gene regulatory networks. In sweet potato, *IbBBX24-IbTOE3-IbPRX17* signaling cascades co-regulate the PRX-mediated ROS-scavenging and crucial role in maintaining the H_2_O_2_ homeostasis in response to multiple abiotic stresses [43]. Another report highlighted the role of the *IbPYL8-IbbHLH66-IbbHLH118* complex in sweet potato in an ABA-dependent fashion under drought stress and enhanced drought tolerance [62]. The mitogen-activated protein kinase (MAPK) signaling pathway plays a crucial role in the signal transduction pathway and is induced by various abiotic and biotic stresses. *IbMPK3/6* enhanced the tolerance against bacterial pathogens and increased the expression of pathogenesis-related genes [79]. *IbMPK3* and *IbMPK6* physically interact and phosphorylate *IbSPF1* (transcriptional regulator during biotic stress signaling), thus playing a crucial role in plant immunity via up-regulating the downstream genes [93] (Fig. 5).

**Figure 6:**
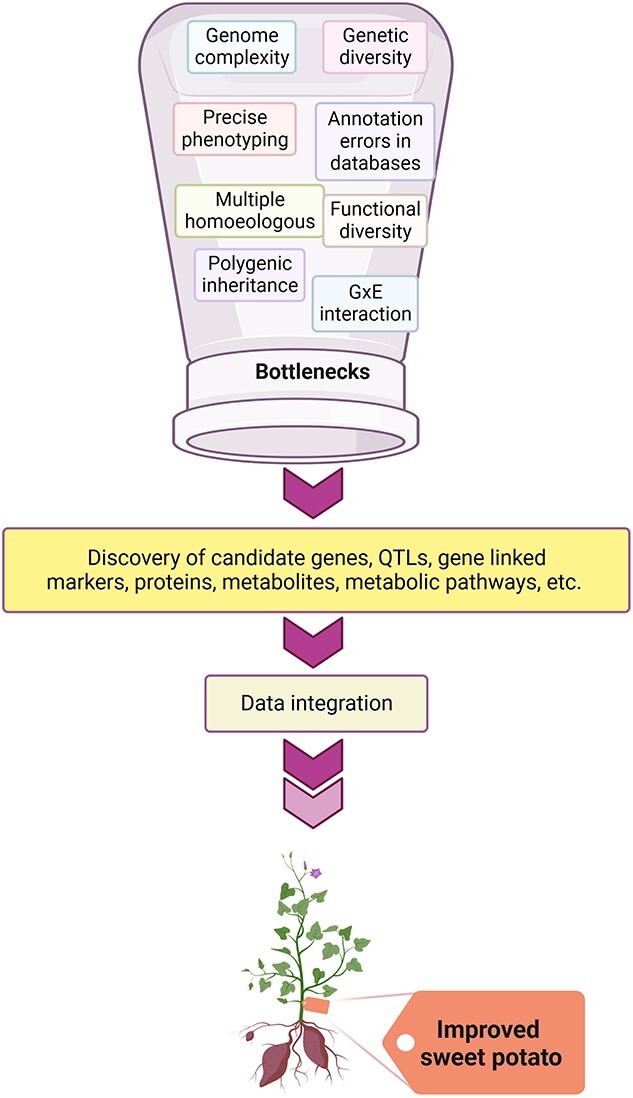
The existing bottlenecks in the utilization of omic approaches to develop stress-resilient sweet potatoes. Modern omics technologies provide a way to overcome the bottlenecks in hexaploid sweet potato breeding via utilizing different molecular/bioinformatics tools, which will help us exploit the new manifesto to develop stress-resilient sweet potato cultivars and assure global food security problems.

The transgenic sweet potato plants expressing the antimicrobial peptide incorporate the resistance against black rot disease caused by *Ceratocystis fimbriata* pathogenic fungus [95]. Another study also elucidated the function of small open reading frames (*sORFs*) in stress responses via functional polypeptides. In sweet potato, *IbEGF* (*sORF* gene) directly interacts with *IbCOP9-5α* to increase drought tolerance via modulating the phytohormone signaling pathways [75]. In plants, sumoylation is a post-translation modification process in which SUMO molecules are attached covalently to the substrate and catalyzed by an enzyme cascade. In sweet potatoes, the SUMO genes *IbSCE1a/b* and *IbSIZ1a/b/c* enhance the drought and salt tolerance, respectively [102]. Furthermore, the roles of other transporters and antiporters were also reported in stress-related responses in sweet potatoes. The OE of *IbSWEET10* (sucrose transporter) in sweet potato increases the resistance against the fungal pathogen *Fusarium oxysporum* via reducing the overall sugar content in transgenic plants [94]. In sweet potato, the higher level of sodium ion compartmentalization into vacuoles also improves the salt and cold stress tolerance and signifies the roles of Na^+^/H^+^ antiporter in sweet potato plant abiotic stress tolerance mechansim [103].

## Role of sweet potato transcriptomics for incorporating stress tolerance

Transcriptomics is the functional map of total mRNA gene expression in a cell/tissue/organism during a definite biological process or stress, which helps to reveal the molecular mechanisms underlying such processes. Transcriptomics studied in non-model crops without a reference genome effectively identifies genes under stress conditions. It also helps understand the molecular mechanisms and provides information about the plant responses under various stresses, thus providing a basis for incorporating stress tolerance via genetic engineering. Several transcriptome analyses were performed in sweet potatoes, allowing us to dissect numerous pathways’ molecular mechanisms during stresses. The first de novo transcript assembly was generated using the diploid and hexaploid sweet potatoes and identified 1661 gene-based microsatellite sequences for drought-stressed conditions [104].

Several other transcriptomics studies were conducted to dissect the molecular mechanism of drought tolerance in sweet potatoes and identified the important genes involved in ABA, ethylene, and JA signaling, drought-inducible TFs such as *bHLH*, *MYB*, *NAC*, *WRKY*, *bZIP*, *HD-ZIP*, thiol specific antioxidant [105], anthocyanin, pentose-phosphate, and photosynthesis biosynthesis pathway [106]. A three-way combined *de novo* transcriptome sequencing in sweet potato identified the positive role of Ca^2+^-ATPase gene in drought tolerance mechanism via promoting ABA singling pathway [107]. The genotypic effect and genotype-specific responses under drought stress conditions were also significant in sweet potatoes [106]. The previous study highlighted the role of several important candidate genes in different drought responses such as leucine-rich repeat (LRR) protein kinases reported roles in dehydration response. The two effectors proteins—light-harvesting chlorophyll A/B-binding-6 (LHCSB6) and slow anion channel-associated-1 (SLAC1)—were found to have roles in the chlorophyll-binding component of PS-II and a guard cell anion efflux protein, respectively [106].

A few studies have also surveyed the global transcriptomic responses under combined abiotic stresses in different *Ipomoea* species. The purple flesh sweet potato (hexaploid) was re-sequenced to dissect the molecular regulation of drought and salinity pathways [108] and depicted the role of glutathione S-transferases family genes which are involved in the accumulation of mono-caffeoylquinic acids to incorporate resistance against abiotic stress [109]. The NBS genes *IbNBS80*, *IbNBS90*, and *IbNBS240* were significantly induced under cold stress, while *IbNBS71*, *IbNBS159*, and *IbNBS208* responded to PEG treatment [53]. Furthermore, the responses of three genes in purple flesh sweet potato were strongly induced (*itb11g07110*, *itb11g02640,* and *itb13g03940*) by drought and oxidative stresses [109]. Guo *et al.* [27] identified several sweet potato NAC TFs (*IbNAC006*, *IbNAC029*, *IbNAC138*, and *IbNAC143*) that have roles under salt stress. Furthermore, a transcriptomics analysis dissected the molecular mechanism of cadmium accumulation in sweet potatoes and shows that cell wall biosynthesis and heavy metal transport pathways are strongly associated with heavy metal toxicity [110]. Another transcriptomics analysis of sweet potato roots under K^+^ deficiency found the crucial roles of hormonal interplay of JA, ethylene, auxin in K^+^ nutrient signaling [111]. The transcriptomic analysis revealed the mechanisms of chilling responses in sweet potato and identified the genes related to carbohydrate metabolism, antioxidant enzymes, hormone metabolism, and cell membrane system under chilling responses [112]. The transcriptomics study of orange-fleshed sweet potato identified the essential secondary metabolic pathways (e.g., carotenoid biosynthesis) involved in stress tolerance and identified important DEGs that participated in metabolite biosynthesis, signal transduction, fatty acid metabolism, and terpenoid backbone biosynthesis [113]. Another report uncovered 55 and 78 different uni-genes involved in the biosynthesis of carotenoid and terpenoid backbone biosynthesis pathways, respectively, via Kyoto Encyclopedia of Genes and Genomes analysis [114]. During the tuber development in sweet potato, an increase in MAPK and calcium signaling-related genes such as nine CDPKs, eight CBLs, and one CaM [115], which were previously reported to have roles in stress responses [116, 117].

Different studies were conducted to identify the key genes involved in disease resistance as well as genes induced by other pathogens in sweet potatoes. The RNA sequencing technology was used to investigate the dynamic changes in root transcriptome profiles at different root development stages after infection with *Fusarium solani* and identified several candidate genes related to plant-pathogen interaction and TFs that could be used to increase the biotic resistance in sweet potato [118]. *De novo* transcriptome assembly and digital gene expression profile of sweet potato identified various differentially expressed genes during defense, such as chitin elicitor receptor kinase-1, MAPK, NAC, WRKY, MYB, ERF TFs, as well as resistance, pathogenesis, SA and JA signaling pathways related genes against *Fusarium* wilt [119]. The combined *de novo* transcriptome assembly also identified the significant and tissue-specific transcripts abundance pattern in seven different tissues of sweet potatoes—plant proteinase inhibitor (kunitz-type protease (sporamins), cysteine protease, and trypsin)—which reported roles in potential stress tolerance, viral genomes and insect resistance [120]. The sweet potato viruses pose a great risk to sweet potato quality and production. Sweet potato feathery mottle virus and sweet potato chlorotic stunt virus are the common viruses in sweet potatoes. To identify the biological mechanism of host responses against these viral pathogens, deep sequencing was performed in the SPVD-susceptible cultivar ‘Beauregard’ upon viral infection. These findings identify several novel responsive elements that can target the NBS-LRR mediated disease resistance genes and involve the downregulation of SA-mediated defense responsive pathway [121]. Another genome-wide study was conducted to identify and characterize the nucleotide-binding site (NBS) encoding genes in a wild ancestor of sweet potatoes (*I. trifida*) and found that four NBS genes (*IbNBS258*, *IbNBS88*, *IbNBS10*, and *IbNBS20*) were significantly induced in response to stem nematode infection, while *IbNBS240*, *IbNBS90*, and *IbNBS80* respond to cold stress, whereas *IbNBS208*, *IbNBS71*, and *IbNBS159* respond to polyethylene glycol (PEG) treatment in sweet potato [53].

## Metabolomics studies in sweet potato for abiotic and biotic stresses

In metabolomics-based crop improvement, the crop phenotype is related to a metabolite; thus, metabolic datasets under stress conditions are significant in understanding the plant responses to stresses. Their corresponding phenotypic information helps improve stress tolerance and adaptability. The metabolomics studies provide comprehensive information regarding the gene’s final products (i.e., metabolites). During abiotic stresses, plant changes the total metabolite profile, which strongly reveals the molecular phenotype of an organism and helps plants under stress adaptation and accumulates a large number of primary and secondary metabolites. Therefore, it is imperative to understand the molecular mechanism of pigmentation in crops. Recently, the integrated metabolic and transcriptional analysis was performed in different flesh colors of sweet potatoes to dissect the role of carotenoid cleavage dioxygenase-4 (*IbCCD4*) in carotenoid disposition during different tuberous root developmental stages [122]. The same approach has been applied to study the molecular mechanisms of anthocyanin and flavonoid accumulation [123]. The OE of sweet potato plants for *IbMYB1* [124] and *IbOr* possessed higher levels of anthocyanins and carotenoids with greater antioxidant activities and showed enhanced tolerance against heat, oxidative [125, 126], and drought stresses [127]. In another report, the same approach was applied and revealed the significant co-annotation of ABA and carotenoid pathway genes and provided helpful information to understand the mechanism governing carotenoid biosynthesis and plant stress mechanism [128]. The sugar molecules act as a significant metabolite in plants that maintain ionic homeostasis, thus retaining structure under stress-induced injury and playing a role in signal transduction mechanisms for abiotic stresses. In sweet potato, the SUT transporter plays a significant role in the molecular regulation of plant stress adaptability via the ABF-dependent ABA signaling pathway [129].

The metabolic engineering in sweet potatoes with low-molecular-weight antioxidants such as carotenoids and vitamins leads to cultivars with enhanced antioxidants and tolerance to abiotic stresses [130]. Furthermore, other metabolic engineering studies in sweet potato *IbGGPS*, *IbZDS*, *IbLCYB2,* and *IbARF5* lead to an increase in the level of carotenoids, such as α-carotene, β-carotene, lutein, β-cryptoxanthin, zeaxanthin and thus incorporate the tolerance against osmotic stress, salt, and drought stresses [46]. RNA interference strategy was also applied to silence the *IbCHY-β* and *IbLCY-ε* genes, which increase the metabolites in sweet potato and enhance tolerance against salt and methyl viologen-mediated oxidative stress, respectively [60]. The metabolite profile of a plant under stress conditions serves as a significant biomarker for plant adaptability and provides a basis for metabolomics-assisted breeding.

## Proteomics: a key for understanding protein structure, function, and regulation under stresses

Proteome analysis elucidates the role of genes related to a specific protein associated with stress [131]. There are relatively few studies on the proteome in sweet potatoes. The comparative proteome and transcriptome analysis between salt-tolerant and salt-sensitive sweet potatoes revealed significant up-regulation of genes involved in stress signaling, plant hormone signal transduction, secondary metabolite accumulation, ion accumulation, redox reactions, and transcriptional regulation. These are the potential pathways to be involved in response to salt stress in sweet potatoes [132]. Furthermore, 93 differentially expressed proteins (DEPs) were found that are specifically expressed in salt-tolerant genotype [132]. In another study, the comparative proteomics analysis highlighted the significant expression level of an extrinsic subunit of PSII (*PsbP*) under heat-stressed IbOr-overexpressing (*At*-OX) *Arabidopsis* plants via directly stabilizing PSII system. The (*At*-OX) *Arabidopsis* plants also showed an increase in carotenoid biosynthesis, thus enhancing the plant’s adaptability under environmental stress [126]. Omics-based biomarkers were developed for identifying sweet potato cultivars by applying the proteomic and metabolomic approaches [133], suggesting that the proteins and metabolites that accumulate differentially may be used as biomarkers to identify stress breeding material for developing new cultivars. The proteogenomic study was performed to functionally characterize the proteomes of sweet potatoes that contribute to map peptides on the haplotype-resolved genome and predicted transcriptome [134]. A recent study reported SUMO system protein’s critical role in salt and drought stress response in sweet potato [102]. Proteomics was also applied to study the disease resistance in sweet potato. The comparative quantitative proteomic analysis was performed to examine the defense mechanisms involved against *Fusarium* wilt in two sweet potato cultivars with differential Fob infection responses. Different proteins related to the signaling transduction pathway, chitinase pathway, subtilisin-like protease, and plant resistance were identified, which depicted significant roles for disease-resistant response. In a nutshell, the omics studies provide a better perspective on precision breeding for developing stress-resilient sweet potatoes and understanding the stress-tolerant mechanism in tuber crops.

## Challenges for stress tolerance in sweet potato and future perspectives

The genetic analysis and breeding of sweet potato has been challenging. Traditional and molecular breeding approaches are used to enhance the tolerance against stresses in sweet potatoes; however, getting novel germplasm with the desirable traits is quite challenging due to various reasons. Firstly, sweet potato is a highly heterozygous hexaploid and posseses a large, complex genome which complicates the inheritance pattern of alleles. Secondly, a large number of possible genotypes are observed in segregating populations because of the diverse combinations of parental chromosomes, which poses a significant challenge to genetic mapping. Lastly, sweet potato plants are almost always self-incompatible and sometimes cross-incompatible. Cross-incompatibility restricts breeding progress when parental lines with desirable traits belong to the same incompatibility group [135]. These factors limit genetic analysis and applications of modern breeding strategies in sweet potato. However, the recent technological advancements in genetic analysis and biotechnology are opening new ways to overcome such challenges in sweet potato genetics (Fig. 6).

The plant’s responses to different abiotic stresses can disturb the equilibrium between energy consumption and energy harvest, thus affecting the overall plant’s growth capacity. Therefore, it is dispensable to gather and process deep information underlying the trade-off mechanisms between plant growth and stress tolerance mechanisms that help to develop crops which can sustain growth and development under adverse environmental conditions. There is also a lack of knowledge about the sweet potato responses to various stress-related environmental stimuli and how these responses coordinate effectively in different cell/tissue types to make long-distance communication.

There is also a need to develop more sophisticated tools to analyse and integrate the vast amount of data generated via the latest sequencing technologies and other omics approaches in sweet potatoes, which can help to draw a better perspective to explore the mechanisms behind biotic and abiotic stresses. The integration of omics data from different apporoaches helps to characterize the dispensable genes and non-coding regions functionally.

Another big challenge plant breeders have recently faced is phenotyping the effects of major stresses and precisely measuring their damage. The commonly used classical ways for evaluating abiotic tolerance are based on the visual scoring system, destructive measurements, and with the help of hand-held equipment to evaluate plant performance under stress. Phenotyping the effects of abiotic stress in crops has conventionally been relatively manual and laborious; however, combining various high-throughput plant phenotyping, such as the latest imaging techniques, machine learning, artificial intelligence etc, could open up new avenues for efficient and cost-effective phenotyping.

The latest genome editing technologies are creating new opportunities for crop improvement through precise genome engineering as transgene-free applications. CRISPR-Cas9 genome-editing technology has been successfully applied in sweetpotato [1], which thus facilitates the development of novel breeding lines for stress tolerances.

## Summary and conclusion

The biotic and environmental stresses are becoming more frequent and long-lasting due to climate change, which seriously threatens global crop production and overall plant development. The plants have developed sophisticated mechanisms to perceive multiple, fluctuating environmental cues and respond precisely. Advanced molecular biology approaches have made it possible to better understand the complex nature of biotic and abiotic stresses, their regulatory networks, and signaling pathways, as well as open new ways to design stress-resilient crops via new breeding programs. This review highlighted the recent omics technologies used in sweet potatoes and gathered knowledge about integrating different technologies to dissect the candidate genes, biosynthetic pathways, and their cross-talk during plant responses under various stressful conditions. The latest omics information helps to understand sweet potato plant functioning and their interactions at the cell and tissue level under adverse environmental conditions. This study will accelerate the characterization of sweet potato plant functional architecture, their responses, and hormonal interplay during biotic and abiotic stress conditions, thus facilitating the progress of genetic improvement in tuber crops. Integrating different omics technologies with conventional breeding approaches paves new ways to incorporate stress tolerance mechanisms in sweet potato, thus ensuring a solution to future food security problems.

## Data Availability

Data sharing is not applicable to this article, as no datasets were generated or analysed during the current study.

## References

[1] Yan M , Nie H, Wang Y. et al. Exploring and exploiting genetics and genomics for sweetpotato improvement: status and perspectives. Plant Comm. 2022;3:100332 10.1016/j.xplc.2022.100332 PMC9482988 35643086

[2] Laveriano-Santos EP , López-Yerena A, Jaime-Rodríguez C. et al. Sweet potato is not simply an abundant food crop: a comprehensive review of its phytochemical constituents, biological activities, and the effects of processing. Antioxidants. 2022;11:1648 36139723 10.3390/antiox11091648 PMC9495970

[3] Tong C , Ru W, Wu L. et al. Fine structure and relationships with functional properties of pigmented sweet potato starches. Food Chem. 2020;311:126011 31862571 10.1016/j.foodchem.2019.126011

[4] Vandenbroucke K , Metzlaff M. Abiotic Stress Tolerant Crops: Genes, Pathways and Bottlenecks. In: Christou P, Savin R, Costa-Pierce BA, Misztal I, Whitelaw CBA, eds. Sustainable Food Production. Springer: New York, 2013,1–17

[5] Park S-U , Lee CJ, Park SC. et al. Flooding tolerance in sweet potato (*Ipomoea batatas* (L.) lam) is mediated by reactive oxygen species and nitric oxide. Antioxidants. 2022;11:878 35624742 10.3390/antiox11050878 PMC9138130

[6] Zhu J-K . Abiotic stress signaling and responses in plants. Cell. 2016;167:313–24 27716505 10.1016/j.cell.2016.08.029 PMC5104190

[7] Fan W , Zhang M, Zhang H. et al. Improved tolerance to various abiotic stresses in transgenic sweet potato (*Ipomoea batatas*) expressing spinach betaine aldehyde dehydrogenase. PLoS One. 2012;7:e37344 22615986 10.1371/journal.pone.0037344 PMC3353933

[8] Khan MA , Gemenet DC, Villordon A. Root system architecture and abiotic stress tolerance: current knowledge in root and tuber crops. Front Plant Sci. 2016;7:01584 10.3389/fpls.2016.01584 PMC5088196 27847508

[9] Kang C , Zhai H, Xue L. et al. A lycopene β-cyclase gene, *IbLCYB2*, enhances carotenoid contents and abiotic stress tolerance in transgenic sweetpotato. Plant Sci. 2018;272:243–54 29807598 10.1016/j.plantsci.2018.05.005

[10] Yang Z , Sun J, Chen Y. et al. Genome-wide identification, structural and gene expression analysis of the bZIP transcription factor family in sweet potato wild relative *Ipomoea trifida*. BMC Genetics. 2019;20:41 31023242 10.1186/s12863-019-0743-y PMC6482516

[11] Voss-Fels KP , Stahl A, Hickey LT. Q&A: modern crop breeding for future food security. BMC Biol. 2019;17:18 30803435 10.1186/s12915-019-0638-4 PMC6390336

[12] Hwang SY , Tseng YT, Lo HF. Application of simple sequence repeats in determining the genetic relationships of cultivars used in sweet potato polycross breeding in Taiwan. Sci Hortic. 2002;93:215–24

[13] Mores A , Borrelli GM, Laidò G. et al. Genomic approaches to identify molecular bases of crop resistance to diseases and to develop future breeding strategies. Int J Mol Sci. 2021;22:5423 34063853 10.3390/ijms22115423 PMC8196592

[14] He LH , Liu X, Liu S. et al. Transcriptomic and targeted metabolomic analysis identifies genes and metabolites involved in anthocyanin accumulation in tuberous roots of sweetpotato (*Ipomoea batatas* L.). Plant Physiol Biochem. 2020;156:323–32 32998099 10.1016/j.plaphy.2020.09.021

[15] Zhang L , Yu Y, Shi T. et al. Genome-wide analysis of expression quantitative trait loci (eQTLs) reveals the regulatory architecture of gene expression variation in the storage roots of sweet potato. Hortic Res. 2020;7:90 32528702 10.1038/s41438-020-0314-4 PMC7261777

[16] Hirakawa H , Okada Y, Tabuchi H. et al. Survey of genome sequences in a wild sweet potato, *Ipomoea trifida* (H. B. K.) G. Don. DNA Res. 2015;22:171–9 25805887 10.1093/dnares/dsv002 PMC4401327

[17] Zhang H , Zhang Q, Zhai H. et al. Transcript profile analysis reveals important roles of jasmonic acid signalling pathway in the response of sweet potato to salt stress. Sci Rep. 2017;7:40819 28084460 10.1038/srep40819 PMC5234020

[18] Si ZZ , Du B, Huo J. et al. A genome-wide BAC-end sequence survey provides first insights into sweetpotato (*Ipomoea batatas* (L.) Lam.) genome composition. BMC Genomics. 2016;17:945 27871234 10.1186/s12864-016-3302-1 PMC5117676

[19] Senthilkumar R , Yeh KW. Multiple biological functions of sporamin related to stress tolerance in sweet potato (*Ipomoea batatas* lam). Biotechnol Adv. 2012;30:1309–17 22306516 10.1016/j.biotechadv.2012.01.022

[20] Wu S , Lau KH, Cao Q. et al. Genome sequences of two diploid wild relatives of cultivated sweetpotato reveal targets for genetic improvement. Nat Commun. 2018;9:4580 30389915 10.1038/s41467-018-06983-8 PMC6214957

[21] Yan L , Lai X, Li X. et al. Analyses of the complete genome and gene expression of chloroplast of sweet potato Ipomoea batata. PLoS One. 2015;10:e0124083 25874767 10.1371/journal.pone.0124083 PMC4398329

[22] Yang J , Moeinzadeh MH, Kuhl H. et al. Haplotype-resolved sweet potato genome traces back its hexaploidization history. Nature Plants. 2017;3:696–703 28827752 10.1038/s41477-017-0002-z

[23] Chen MJ , Fan W, Ji F. et al. Genome-wide identification of agronomically important genes in outcrossing crops using OutcrossSeq. Mol Plant. 2021;14:556–70 33429094 10.1016/j.molp.2021.01.003

[24] Xiao SZ , Dai X, Zhao L. et al. Resequencing of sweetpotato germplasm resources reveals key loci associated with multiple agronomic traits. Hortic Res. 2023;10:uhac234 36643760 10.1093/hr/uhac234 PMC9832839

[25] Nie N , Huo J, Sun S. et al. Genome-wide characterization of the PIFs family in sweet potato and functional identification of IbPIF3.1 under drought and Fusarium wilt stresses. Int J Mol Sci. 2023;24:4092 36835500 10.3390/ijms24044092 PMC9965949

[26] Si Z , Wang L, Ji Z. et al. Genome-wide comparative analysis of the valine glutamine motif containing genes in four ipomoea species. BMC Plant Biol. 2023;23:209 37085761 10.1186/s12870-023-04235-6 PMC10122360

[27] Guo F , Liu S, Zhang C. et al. Genome-wide systematic survey and analysis of NAC transcription factor family and their response to abiotic stress in sweetpotato. Sci Hortic. 2022;299:111048

[28] Medison MB , Pan R, Peng Y. et al. Identification of HQT gene family and their potential function in CGA synthesis and abiotic stresses tolerance in vegetable sweet potato. Physiol Mol Biol Plants. 2023;29:361–76 37033766 10.1007/s12298-023-01299-4 PMC10073390

[29] Zhang CB , Dong T, Yu J. et al. Genome-wide survey and expression analysis of Dof transcription factor family in sweetpotato shed light on their promising functions in stress tolerance. Front Plant Sci. 2023;14:1140727 36895872 10.3389/fpls.2023.1140727 PMC9989284

[30] Si ZZ , Wang L, Ji Z. et al. Comparative analysis of the MYB gene family in seven Ipomoea species. Front Plant Sci. 2023;14:1155018 37021302 10.3389/fpls.2023.1155018 PMC10067929

[31] Liu EL , Li Z, Luo Z. et al. Genome-wide identification of DUF668 gene family and expression analysis under drought and salt stresses in sweet potato *Ipomoea batatas* (L.) lam. Genes. 2023;14:217 36672958 10.3390/genes14010217 PMC9858669

[32] Huo RX , Zhao Y, Liu T. et al. Genome-wide identification and expression analysis of two-component system genes in sweet potato (*Ipomoea batatas* L.). Front Plant Sci. 2023;13:1091620 36714734 10.3389/fpls.2022.1091620 PMC9878860

[33] Zhang JZ , He PW, Xu XM. et al. Genome-wide identification and expression analysis of the xyloglucan endotransglucosylase/hydrolase gene family in sweet potato *Ipomoea batatas* (L.) lam. Int J Mol Sci. 2023;24:775 36614218 10.3390/ijms24010775 PMC9820959

[34] Li M , Chen L, Lang T. et al. Genome-wide identification and expression analysis of Expansin gene family in the storage root development of diploid wild sweetpotato *Ipomoea trifida*. Genes. 2022;13:1043 35741805 10.3390/genes13061043 PMC9222398

[35] Lu Y , Sun J, Yang Z. et al. Genome-wide identification and expression analysis of glycine-rich RNA-binding protein family in sweet potato wild relative *Ipomoea trifida*. Gene. 2019;686:177–86 30453066 10.1016/j.gene.2018.11.044

[36] Liu SY , Zhang C, Guo F. et al. A systematical genome-wide analysis and screening of WRKY transcription factor family engaged in abiotic stress response in sweetpotato. BMC Plant Biol. 2022;22:616 36575404 10.1186/s12870-022-03970-6 PMC9795774

[37] Xie H , Yang Q, Wang X. et al. Genome-wide identification of the A20/AN1 zinc finger protein family genes in *Ipomoea batatas* and its two relatives and function analysis of IbSAP16 in salinity tolerance. 2022;23:11551 10.3390/ijms231911551 PMC9570247 36232853

[38] He S , Hao X, He S. et al. Genome-wide identification, phylogeny and expression analysis of AP2/ERF transcription factors family in sweet potato. BMC Genomics. 2021;22:748 34656106 10.1186/s12864-021-08043-w PMC8520649

[39] Huang ZW , Wang Z, Li X. et al. Genome-wide identification and expression analysis of JAZ family involved in hormone and abiotic stress in sweet potato and its two diploid relatives. Int J Mol Sci. 2021;22:9786 34575953 10.3390/ijms22189786 PMC8468994

[40] Zhuang J , Zhang J, Hou XL. et al. Transcriptomic, proteomic, metabolomic and functional genomic approaches for the study of abiotic stress in vegetable crops. Crit Rev Plant Sci. 2014;33:225–37

[41] Meng X , Liu S, Zhang C. et al. The unique sweet potato NAC transcription factor IbNAC3 modulates combined salt and drought stresses. Plant Physiol. 2023;191:747–71 36315103 10.1093/plphys/kiac508 PMC9806649

[42] He ST , Wang H, Hao X. et al. Dynamic network biomarker analysis discovers IbNAC083 in the initiation and regulation of sweet potato root tuberization. Plant J. 2021;108:793–813 34460981 10.1111/tpj.15478

[43] Zhang H , Wang Z, Li X. et al. The IbBBX24–IbTOE3–IbPRX17 module enhances abiotic stress tolerance by scavenging reactive oxygen species in sweet potato. New Phytol. 2022;233:1133–52 34773641 10.1111/nph.17860

[44] Zhu H , Zhai H, He S. et al. A novel sweetpotato GATA transcription factor, IbGATA24, interacting with IbCOP9-5a positively regulates drought and salt tolerance. Environ Exp Bot. 2022;194:104735

[45] Wang W , Qiu X, Yang Y. et al. Sweetpotato bZIP transcription factor *IbABF4* confers tolerance to multiple abiotic stresses. Front Plant Sci. 2019;10:630:10.3389/fpls.2019.00630 PMC6531819 31156685

[46] Kang C , He S, Zhai H. et al. A sweetpotato auxin response factor gene (IbARF5) is involved in carotenoid biosynthesis and salt and drought tolerance in transgenic Arabidopsis. Front Plant Sci. 2018;9:1307 30254657 10.3389/fpls.2018.01307 PMC6141746

[47] Li Y , Zhang H, Zhang Q. et al. An AP2/ERF gene, *IbRAP2-12*, from sweetpotato is involved in salt and drought tolerance in transgenic *Arabidopsis*. Plant Sci. 2019;281:19–30 30824052 10.1016/j.plantsci.2019.01.009

[48] Zhao H , Zhao H, Hu Y. et al. Expression of the sweet potato MYB transcription factor IbMYB48 confers salt and drought tolerance in Arabidopsis. Genes. 2022;13:1883 36292768 10.3390/genes13101883 PMC9602379

[49] Kim S-E , Lee CJ, Ji CY. et al. Transgenic sweetpotato plants overexpressing *tocopherol cyclase* display enhanced α-tocopherol content and abiotic stress tolerance. Plant Physiol Biochem. 2019;144:436–44 31639559 10.1016/j.plaphy.2019.09.046

[50] Ke Q , Kang L, Kim HS. et al. Down-regulation of *lycopene ε-cyclase* expression in transgenic sweetpotato plants increases the carotenoid content and tolerance to abiotic stress. Plant Sci. 2019;281:52–60 30824061 10.1016/j.plantsci.2019.01.002

[51] Meng X , Li G, Yu J. et al. Isolation, expression analysis, and function evaluation of 12 novel stress-responsive genes of NAC transcription factors in sweetpotato. Crop Sci. 2018;58:1328–41

[52] Chen SP , Kuo CH, Lu HH. et al. The sweet potato NAC-domain transcription factor IbNAC1 is dynamically coordinated by the activator IbbHLH3 and the repressor IbbHLH4 to reprogram the defense mechanism against wounding. PLoS Genet. 2016;12:e1006397 27780204 10.1371/journal.pgen.1006397 PMC5079590

[53] Si ZZ , Qiao YK, Zhang K. et al. Characterization of nucleotide binding -site-encoding genes in sweetpotato, *Ipomoea batatas* (L.) lam., and their response to biotic and abiotic stresses. Cytogenet Genome Res. 2021;161:257–71 34320507 10.1159/000515834

[54] Yu T , Zhou HA, Liu ZL. et al. The sweet potato transcription factor IbbHLH33 enhances chilling tolerance in transgenic tobacco. Czech J Genet Plant Breed. 2022;58:210–22

[55] Zhang C , Liu S, Liu D. et al. Genome-wide survey and expression analysis of GRAS transcription factor family in sweetpotato provides insights into their potential roles in stress response. BMC Plant Biol. 2022;22:232 35524176 10.1186/s12870-022-03618-5 PMC9074257

[56] Noh SA , Park SH, Huh GH. et al. Growth retardation and differential regulation of expansin genes in chilling-stressed sweetpotato. Plant Biotech Rep. 2009;3:75–85

[57] Wang H , Yang J, Zhang M. et al. Altered phenylpropanoid metabolism in the maize Lc-expressed sweet potato (*Ipomoea batatas*) affects storage root development. Sci Rep. 2016;6:18645 26727353 10.1038/srep18645 PMC4698713

[58] Xing SH , Li R, Zhao H. et al. The transcription factor IbNAC29 positively regulates the carotenoid accumulation in sweet potato. Hortic Res. 2023;10:uhad010 36960431 10.1093/hr/uhad010 PMC10028406

[59] Goo Y-M , Han EH, Jeong JC. et al. Overexpression of the sweet potato IbOr gene results in the increased accumulation of carotenoid and confers tolerance to environmental stresses in transgenic potato. Comptes Rendus Biologies. 2015;338:12–20 25528672 10.1016/j.crvi.2014.10.006

[60] Kim SH , Kim YH, Ahn YO. et al. Downregulation of the lycopene ε-cyclase gene increases carotenoid synthesis via the β-branch-specific pathway and enhances salt-stress tolerance in sweetpotato transgenic calli. Physiol Plant. 2013;147:432–42 22938023 10.1111/j.1399-3054.2012.01688.x

[61] Wang H , Fan W, Li H. et al. Functional characterization of Dihydroflavonol-4-reductase in anthocyanin biosynthesis of purple sweet potato underlies the direct evidence of anthocyanins function against abiotic stresses. PLoS One. 2013;8:e78484 24223813 10.1371/journal.pone.0078484 PMC3817210

[62] Xue LY , Wei Z, Zhai H. et al. The IbPYL8-IbbHLH66-IbbHLH118 complex mediates the abscisic acid-dependent drought response in sweet potato. New Phytol. 2022;236:2151–71 36128653 10.1111/nph.18502

[63] Wang DD , Liu HJ, Wang HX. et al. A novel sucrose transporter gene IbSUT4 involves in plant growth and response to abiotic stress through the ABF-dependent ABA signaling pathway in Sweetpotato. BMC Plant Biol. 2020;20:157 32293270 10.1186/s12870-020-02382-8 PMC7157994

[64] Zhai H , Wang F, Si Z. et al. A myo-inositol-1-phosphate synthase gene, IbMIPS1, enhances salt and drought tolerance and stem nematode resistance in transgenic sweet potato. Plant Biotechnol J. 2016;14:592–602 26011089 10.1111/pbi.12402 PMC11389020

[65] Kwak S-S , Huan Y, Xiaoyun J. et al. Isolation, expression and function analysis of a bZIP transcription factor IbbZIP37 in sweetpotato (*Ipomoea batatas* L. [lam]). Emirates J Food Agric. 2019;31:134–42

[66] Liu DG , He S, Zhai H. et al. Overexpression of IbP5CR enhances salt tolerance in transgenic sweetpotato. Plant Cell Tissue Org Cult. 2014;117:1–16

[67] Liu D , Wang L, Zhai H. et al. A novel α/β-hydrolase gene IbMas enhances salt tolerance in transgenic sweetpotato. PLoS One. 2014;9:e115128 25501819 10.1371/journal.pone.0115128 PMC4264881

[68] Yu YC , Xuan Y, Bian X. et al. Overexpression of phosphatidylserine synthase IbPSS1 affords cellular Na+ homeostasis and salt tolerance by activating plasma membrane Na+/H+ antiport activity in sweet potato roots. Hortic Res. 2020;7:131 32821414 10.1038/s41438-020-00358-1 PMC7395154

[69] Wang C , Wang L, Lei J. et al. IbMYB308, a sweet potato R2R3-MYB gene, improves salt stress tolerance in transgenic tobacco. Genes. 2022;13:1476 36011387 10.3390/genes13081476 PMC9408268

[70] Munns R , Tester M. Mechanisms of salinity tolerance. Annu Rev Plant Biol. 2008;59:651–81 18444910 10.1146/annurev.arplant.59.032607.092911

[71] Sun J , Wang M-J, Ding M-Q. et al. H_2_O_2_ and cytosolic Ca^2+^ signals triggered by the PM H^+^-coupled transport system mediate K^+^/Na^+^ homeostasis in NaCl-stressed Populus euphratica cells. Plant Cell Environ. 2010;33:943–58 20082667 10.1111/j.1365-3040.2010.02118.x

[72] You C , Li C, Ma M. et al. A C2-domain Abscisic acid-related gene, *IbCAR1*, positively enhances salt tolerance in sweet potato (*Ipomoea batatas* (L.) lam.). Int J Mol Sci. 2022;23:9680 36077077 10.3390/ijms23179680 PMC9456122

[73] Du B , Nie N, Sun S. et al. A novel sweetpotato RING-H2 type E3 ubiquitin ligase gene IbATL38 enhances salt tolerance in transgenic Arabidopsis. Plant Sci. 2021;304:110802 33568301 10.1016/j.plantsci.2020.110802

[74] Bian X , Kim HS, Kwak SS. et al. Different functions of *IbRAP2.4*, a drought-responsive AP2/ERF transcription factor, in regulating root development between *Arabidopsis* and Sweetpotato. Front Plant Sci. 2022;13:820450 35154229 10.3389/fpls.2022.820450 PMC8826056

[75] Zhou Y , Zhai H, Xing S. et al. A novel small open reading frame gene, IbEGF, enhances drought tolerance in transgenic sweet potato. Front Plant Sci. 2022;13:965069 36388596 10.3389/fpls.2022.965069 PMC9660231

[76] Zhang H , Zhang Q, Zhai H. et al. IbBBX24 promotes the jasmonic acid pathway and enhances Fusarium wilt resistance in sweet potato. Plant Cell. 2020;32:1102–23 32034034 10.1105/tpc.19.00641 PMC7145486

[77] Wang W , Yu H, Kim HS. et al. Molecular characterization of a sweet potato stress tolerance-associated trehalose-6-phosphate synthase 1 gene (*IbTPS1*) in response to abiotic stress. Plant Biotechnol Rep. 2019;13:235–43

[78] Park S-C , Kang L, Park WS. et al. Carotenoid cleavage dioxygenase 4 (CCD4) cleaves β-carotene and interacts with IbOr in sweetpotato. Plant Biotechnol Rep. 2020;14:737–42

[79] Kim HS , Park SC, Ji CY. et al. Molecular characterization of biotic and abiotic stress-responsive MAP kinase genes, IbMPK3 and IbMPK6, in sweetpotato. Plant Physiol Biochem. 2016;108:37–48 27404133 10.1016/j.plaphy.2016.06.036

[80] Zhu H , Zhou Y, Zhai H. et al. A novel Sweetpotato WRKY transcription factor, IbWRKY2, positively regulates drought and salt tolerance in transgenic Arabidopsis. Biomol Ther. 2020;10:506 10.3390/biom10040506 PMC7226164 32230780

[81] Wang F-B , Zhai H, An Y. et al. Overexpression of IbMIPS1 gene enhances salt tolerance in transgenic sweetpotato. J Integr Agric. 2016;15:271–81

[82] Kim Y-H , Kim HS, Park SC. et al. Downregulation of *swpa4* peroxidase expression in transgenic sweetpotato plants decreases abiotic stress tolerance and reduces stress-related peroxidase expression. Plant Biotechnol Rep. 2021;15:69–76

[83] Jiang T , Zhai H, Wang F. et al. Cloning and characterization of a salt tolerance-associated gene encoding Trehalose-6-phosphate synthase in Sweetpotato. J Integr Agric. 2014;13:1651–61

[84] Yang D , Xie Y, Sun H. et al. IbINH positively regulates drought stress tolerance in sweetpotato. Plant Physiol Biochem. 2020;146:403–10 31794900 10.1016/j.plaphy.2019.11.039

[85] Jin R , Kim HS, Yu T. et al. Identification and function analysis of bHLH genes in response to cold stress in sweetpotato. Plant Physiol Biochem. 2021;169:224–35 34808465 10.1016/j.plaphy.2021.11.027

[86] Lee C-J , Park SU, Kim SE. et al. Overexpression of *IbLfp* in sweetpotato enhances the low-temperature storage ability of tuberous roots. Plant Physiol Biochem. 2021;167:577–85 34461554 10.1016/j.plaphy.2021.08.041

[87] Lee C-J , Kim SE, Park SU. et al. Overexpression of *IbFAD8* enhances the low-temperature storage ability and alpha-linolenic acid content of sweetpotato tuberous roots. Front Plant Sci. 2021;12:764100 34777447 10.3389/fpls.2021.764100 PMC8589035

[88] Kim S-E , Lee CJ, Park SU. et al. Overexpression of the golden SNP-carrying *Orange* gene enhances carotenoid accumulation and heat stress tolerance in Sweetpotato plants. Antioxidants. 2021;10:51 33406723 10.3390/antiox10010051 PMC7823567

[89] Liu X , Wang Y, Zhu H. et al. Natural allelic variation confers high resistance to sweet potato weevils in sweet potato. Nature Plants. 2022;8:1233–44 36376755 10.1038/s41477-022-01272-1

[90] Liao Y , Zeng L, Rao S. et al. Induced biosynthesis of chlorogenic acid in sweetpotato leaves confers the resistance against sweetpotato weevil attack. J Adv Res. 2020;24:513–22 32612857 10.1016/j.jare.2020.06.011 PMC7320233

[91] Nokihara K , Okada Y, Ohata S. et al. Transcriptome analysis reveals key genes involved in weevil resistance in the hexaploid sweetpotato. Plants. 2021;10:1535 34451581 10.3390/plants10081535 PMC8398197

[92] Zhong YY , Ahmed S, Deng G. et al. Improved insect resistance against Spodoptera litura in transgenic sweetpotato by overexpressing Cry1Aa toxin. Plant Cell Rep. 2019;38:1439–48 31451933 10.1007/s00299-019-02460-8

[93] Kim HS , Bian X, Lee CJ. et al. IbMPK3/IbMPK6-mediated IbSPF1 phosphorylation promotes tolerance to bacterial pathogen in sweetpotato. Plant Cell Rep. 2019;38:1403–15 31367772 10.1007/s00299-019-02451-9

[94] Li Y , Wang Y, Zhang H. et al. The plasma membrane-localized sucrose transporter IbSWEET10 contributes to the resistance of sweet potato to *Fusarium oxysporum*. Front Plant Sci. 2017;8:197 28261250 10.3389/fpls.2017.00197 PMC5306249

[95] Muramoto N , Tanaka T, Shimamura T. et al. Transgenic sweet potato expressing thionin from barley gives resistance to black rot disease caused by *Ceratocystis fimbriata* in leaves and storage roots. Plant Cell Rep. 2012;31:987–97 22212462 10.1007/s00299-011-1217-5

[96] Okada Y , Saito A, Nishiguchi M. et al. Virus resistance in transgenic sweetpotato [*Ipomoea batatas* L. (lam)] expressing the coat protein gene of sweet potato feathery mottle virus. Theor Appl Genet. 2001;103:743–51

[97] Sivparsad BJ , Gubba A. Development of transgenic sweet potato with multiple virus resistance in South Africa (SA). Transgenic Res. 2014;23:377–88 24158330 10.1007/s11248-013-9759-7

[98] Mwanga ROM , Kriegner A, Cervantes-Flores JC. et al. Resistance to Sweetpotato chlorotic stunt virus and Sweetpotato feathery mottle virus is mediated by two separate recessive genes in sweetpotato. J Am Soc Hortic Sc JASHS. 2002;127:806

[99] Kreuze JF , Klein IS, Lazaro MU. et al. RNA silencing-mediated resistance to a crinivirus (Closteroviridae) in cultivated sweetpotato (*Ipomoea batatas* L.) and development of sweetpotato virus disease following co-infection with a potyvirus. Mol Plant Pathol. 2008;9:589–98 19018990 10.1111/j.1364-3703.2008.00480.x PMC6640417

[100] Ojaghian S , Wang L, Xie G-L. Enhanced resistance to white rot in *Ipomoea batatas* expressing a *Trichoderma harzianum* chitinase gene. J Gen Plant Pathol. 2020;86:412–8

[101] Yu Y , Pan Z, Wang X. et al. Targeting of SPCSV-*RNase3* via CRISPR-Cas13 confers resistance against sweet potato virus disease. Mol Plant Pathol. 2022;23:104–17 34633749 10.1111/mpp.13146 PMC8659606

[102] Zhang Y , Lyu S, Hu Z. et al. Identification and functional characterization of the SUMO system in sweet potato under salt and drought stress. Plant Sci. 2023;330:111645 36828141 10.1016/j.plantsci.2023.111645

[103] Fan W , Deng G, Wang H. et al. Elevated compartmentalization of Na+ into vacuoles improves salt and cold stress tolerance in sweet potato (*Ipomoea batatas*). Physiol Plant. 2015;154:560–71 25307930 10.1111/ppl.12301

[104] Schafleitner R , Tincopa LR, Palomino O. et al. A sweetpotato gene index established by de novo assembly of pyrosequencing and sanger sequences and mining for gene-based microsatellite markers. BMC Genomics. 2010;11:604 20977749 10.1186/1471-2164-11-604 PMC3017860

[105] Zhu H , Zhou Y, Zhai H. et al. Transcriptome profiling reveals insights into the molecular mechanism of drought tolerance in sweetpotato. J Integr Agric. 2019;18:9–23

[106] Lau KH , del Rosario Herrera M, Crisovan E. et al. Transcriptomic analysis of sweet potato under dehydration stress identifies candidate genes for drought tolerance. Plant Direct. 2018;2:e00092 31245692 10.1002/pld3.92 PMC6508841

[107] Yang YF , Wang Y, Jia L. et al. Involvement of an ABI-like protein and a Ca2+-ATPase in drought tolerance as revealed by transcript profiling of a sweetpotato somatic hybrid and its parents *Ipomoea batatas* (L.) lam. and I-triloba L. PLoS One. 2018;13:e0193193 29466419 10.1371/journal.pone.0193193 PMC5821372

[108] Arisha MH , Aboelnasr H, Ahmad MQ. et al. Transcriptome sequencing and whole genome expression profiling of hexaploid sweetpotato under salt stress. BMC Genomics. 2020;21:197 32131729 10.1186/s12864-020-6524-1 PMC7057664

[109] Soviguidi DRJ , Liu Y, Pan R. et al. Role of sweet potato GST genes in abiotic stress tolerance revealed by genomic and transcriptomic analyses. Crop Breed Applied Biotechnol. 2022;22:e36852212

[110] Yin AG , Shen C, Huang Y. et al. Transcriptomic analyses of sweet potato in response to cd exposure and protective effects of K on Cd-induced physiological alterations. Environ Sci Pollut Res. 2022;29:36824–38 10.1007/s11356-021-18144-4 35064501

[111] Wang F , Tan W-F, Song W. et al. Transcriptome analysis of sweet potato responses to potassium deficiency. BMC Genomics. 2022;23:655 36109727 10.1186/s12864-022-08870-5 PMC9479357

[112] Xie ZY , Zhou Z, Li H. et al. High throughput sequencing identifies chilling responsive genes in sweetpotato (*Ipomoea batatas* lam.) during storage. Genomics. 2019;111:1006–17 29792923 10.1016/j.ygeno.2018.05.014

[113] Li RJ , Zhai H, Kang C. et al. De novo transcriptome sequencing of the orange-fleshed sweet potato and analysis of differentially expressed genes related to carotenoid biosynthesis. Int J Genom. 2015;2015:843802 10.1155/2015/843802 PMC4663004 26649293

[114] Qin Z , Li A, Hou F. et al. Gene identification using RNA-seq in two sweetpotato genotypes and the use of mining to analyze carotenoid biosynthesis. S Afr J Bot. 2017;109:189–95

[115] Cai ZQ , Cai Z, Huang J. et al. Transcriptomic analysis of tuberous root in two sweet potato varieties reveals the important genes and regulatory pathways in tuberous root development. BMC Genomics. 2022;23:473 35761189 10.1186/s12864-022-08670-x PMC9235109

[116] Chen T , Wu X, Chen Y. et al. Combined proteomic and cytological analysis of Ca2^+^-calmodulin regulation in *Picea meyeri* pollen tube growth. Plant Physiol. 2009;149:1111–26 19011005 10.1104/pp.108.127514 PMC2633844

[117] Hirt H . Connecting oxidative stress, auxin, and cell cycle regulation through a plant mitogen-activated protein kinase pathway. Proc Natl Acad Sci U S A. 2000;97:2405–7 10716978 10.1073/pnas.97.6.2405 PMC33972

[118] Zhang C , Luo Q, Tang W. et al. Transcriptome characterization and gene changes induced by *Fusarium solani* in sweetpotato roots. Genes. 2023;14:969 37239329 10.3390/genes14050969 PMC10218436

[119] Lin YL , Zou W, Lin S. et al. Transcriptome profiling and digital gene expression analysis of sweet potato for the identification of putative genes involved in the defense response against *Fusarium oxysporum* f. sp batatas. PLoS One. 2017;12:e0187838 29131830 10.1371/journal.pone.0187838 PMC5683638

[120] Tao X , Gu YH, Wang HY. et al. Digital gene expression analysis based on integrated de novo transcriptome assembly of sweet potato *Ipomoea batatas* (L.) lam. PLoS One. 2012;7:e36234 22558397 10.1371/journal.pone.0036234 PMC3338685

[121] Bednarek R , David M, Fuentes S. et al. Transcriptome analysis provides insights into the responses of sweet potato to sweet potato virus disease (SPVD). Virus Res. 2021;295:198293 33412165 10.1016/j.virusres.2020.198293 PMC7985617

[122] Zhang J , He L, Dong J. et al. Integrated metabolic and transcriptional analysis reveals the role of carotenoid cleavage dioxygenase 4 (IbCCD4) in carotenoid accumulation in sweetpotato tuberous roots. Biotech Biofuel Bioprod. 2023;16:45 10.1186/s13068-023-02299-y PMC10012543 36918944

[123] Zhao DL , Zhao L, Liu Y. et al. Metabolomic and transcriptomic analyses of the flavonoid biosynthetic pathway for the accumulation of anthocyanins and other flavonoids in sweetpotato root skin and leaf vein base. J Agric Food Chem. 2022;70:2574–88 35175040 10.1021/acs.jafc.1c05388

[124] Park SC , Kim YH, Kim SH. et al. Overexpression of the IbMYB1 gene in an orange-fleshed sweet potato cultivar produces a dual-pigmented transgenic sweet potato with improved antioxidant activity. Physiol Plant. 2015;153:525–37 25220246 10.1111/ppl.12281

[125] Park SC , Kim SH, Park S. et al. Enhanced accumulation of carotenoids in sweetpotato plants overexpressing IbOr-ins gene in purple-fleshed sweetpotato cultivar. Plant Physiol Biochem. 2015;86:82–90 25438140 10.1016/j.plaphy.2014.11.017

[126] Kang L , Kim HS, Kwon YS. et al. IbOr regulates photosynthesis under heat stress by stabilizing IbPsbP in sweetpotato. Front Plant Sci. 2017;8:989 28642783 10.3389/fpls.2017.00989 PMC5462972

[127] Kim HS , Ji CY, Lee CJ. et al. Orange: a target gene for regulating carotenoid homeostasis and increasing plant tolerance to environmental stress in marginal lands. J Exp Bot. 2018;69:3393–400 29385615 10.1093/jxb/ery023

[128] Ren Q , Zhen X, Gao H. et al. Integrated metabolomic and transcriptomic analyses reveal the basis for carotenoid biosynthesis in sweet potato (*Ipomoea batatas* (L.) lam.) storage roots. Metabolites. 2022;12:1010 36355093 10.3390/metabo12111010 PMC9699360

[129] Saddhe AA , Manuka R, Penna S. Plant sugars: homeostasis and transport under abiotic stress in plants. Physiol Plant. 2021;171:739–55 33215734 10.1111/ppl.13283

[130] Kang L , Park SC, Ji CY. et al. Metabolic engineering of carotenoids in transgenic sweetpotato. Breed Sci. 2017;67:27–34 28465665 10.1270/jsbbs.16118 PMC5407916

[131] Das P , Nutan KK, Singla-Pareek SL. et al. Understanding salinity responses and adopting 'omics-based' approaches to generate salinity tolerant cultivars of rice. Front Plant Sci. 2015;6:712 26442026 10.3389/fpls.2015.00712 PMC4563168

[132] Meng XQ , Liu S, Dong T. et al. Comparative transcriptome and proteome analysis of salt-tolerant and salt-sensitive sweet potato and overexpression of IbNAC7 confers salt tolerance in Arabidopsis. Front Plant Sci. 2020;11:572540 32973858 10.3389/fpls.2020.572540 PMC7481572

[133] Lee SJ , Kim JY, Kim YC. et al. Omics-based biomarkers for the identification of six Korean cultivars of sweet potato (*Ipomoea batatas* L. lam). J Hortic Sci Biotechnol. 2013;88:509–18

[134] Al-Mohanna T , Ahsan N, Bokros NT. et al. Proteomics and proteogenomics analysis of sweetpotato (*Ipomoea batatas*) leaf and root. J Proteome Res. 2019;18:2719–34 31117636 10.1021/acs.jproteome.8b00943

[135] Dufresne F , Stift M, Vergilino R. et al. Recent progress and challenges in population genetics of polyploid organisms: an overview of current state-of-the-art molecular and statistical tools. Mol Ecol. 2014;23:40–69 24188632 10.1111/mec.12581

